# How could climate change influence the distribution of the black soldier fly, *Hermetiaillucens* (Linnaeus) (Diptera, Stratiomyidae)?

**DOI:** 10.3897/BDJ.10.e90146

**Published:** 2022-10-17

**Authors:** Alex Pazmiño-Palomino, Carolina Reyes-Puig, Ana G. Del Hierro

**Affiliations:** 1 Instituto Nacional de Biodiversidad, Quito, Ecuador Instituto Nacional de Biodiversidad Quito Ecuador; 2 Colegio de Ciencias Biológicas y Ambientales COCIBA, Museo de Zoología & Laboratorio de Zoología Terrestre, Instituto iBIOTROP, Universidad San Francisco de Quito USFQ, Quito, 170901, Ecuador Colegio de Ciencias Biológicas y Ambientales COCIBA, Museo de Zoología & Laboratorio de Zoología Terrestre, Instituto iBIOTROP, Universidad San Francisco de Quito USFQ Quito, 170901 Ecuador

**Keywords:** BSF, citizen science, *Hermetiaillucens*, iNaturalist, species distribution model

## Abstract

The black soldier fly, *Hermetiaillucens* (Linnaeus, 1758), is a saprophagous species used to decompose organic matter. This study proposes a distribution model of *H.illucens* to illustrate its current and future distribution. The methodology includes data collection from the Global Biodiversity Information Facility (GBIF), complemented with iNaturalist, manual expert curation of occurrence records, six species distribution models algorithms and one ensemble model. The average temperature of the driest annual quarter and the precipitation of the coldest annual quarter were the key variables influencing the potential distribution of *H.illucens*. The distribution range is estimated to decrease progressively and their suitable habitats could change dramatically in the future due to global warming. On the other hand, current optimal habitats would become uninhabitable for the species, mainly at low latitudes. Under this scenario, the species is projected to move to higher latitudes and elevations in the future. The results of this study provide data on the distribution of *H.illucens*, facilitating its location, management and sustainable use in current and future scenarios.

## Introduction

Insects (Insecta) are amongst the most diverse and efficient groups on the planet, capable of performing ecological functions at different levels of food chains (Price et al. 2011, Stork 2018). One of this class´ prominent roles is the decomposition of organic matter, the ability to reintroduce nutrients into food chains, closing of biogeochemical cycles (Pechal et al. 2014). Within more than 150,000 known species in the Diptera order, a considerable number of taxa have been studied for their association with organic decomposition (Morales Mira and Peláez Jaramillo 2010, Marshall et al. 2015, Mertenat et al. 2019). The black soldier fly (BSF), *Hermetiaillucens* (Linnaeus, 1758), is the best-known Diptera species for its strict relationship with decomposing organic matter (Siddiqui et al. 2022). Worldwide, the BSF is applied for its outstanding conversion efficiency, which is why this species is bred in captivity around the world (Wang and Shelomi 2017). Under optimal conditions, BSF larvae consume from 25 to 500 milligrams of organic matter per individual per day (Bava et al. 2019, Surendra et al. 2020). Regardless of this insect's great economic importance, there is an apparent absence of updated information on the global distribution and possible environmental factors that model its distribution (Müller et al. 2017). Therefore, researchers, decision-makers, waste managers, livestock producers, farmers and others urgently need information about *H.illucens* distribution to plan future environmental projects.

*Hermetiaillucens*, very likely native to the tropics and subtropics of the Neotropical Region (Kaya et al. 2021), is a member of the subfamily Hermetiinae (Diptera: Stratiomyidae). With 42 species in the Neotropical Region, 21 in the Oriental, 11 in Nearctic and 10 in the Australian, the genus *Hermetia* Latreille has the most significant number of species amongst the genera of the subfamily (Lessard et al. 2019) and the most extensive global distribution within the subfamily Hermetiinae (Woodley 2001, Hauser et al. 2017, Fachin and Carvalho-Filho 2020, Ivorra et al. 2020, Fachin and Hauser 2022). Specifically, *Hermetiaillucens* is the species with thewidest distribution within this fly family (Woodley 2001, Roháček and Hora 2013, Marshall et al. 2015,Lessard et al. 2019). However, its distribution is a topic of debate and research that could be useful for sustainable use and protecting current ecosystems for this species. Scientific evidence points to global climate change causing abiotic factors affecting species distribution worldwide (Walther et al. 2002). Consequently, it is crucial to know how climate change would influence the distribution of these species. Ecological niche models (ENMs) are practical tools for predicting the suitability of the environment for the species, based on the ecological and geographical space they occupy (Elith et al. 2006, Lee et al. 2021). ENM can be used with different approaches, from endemic, invasive species, pests, medical or economic importance, diverse conservation purposes, sustainable use and control, according to the case (Phillips et al. 2006, Moo-Llanes et al. 2013, Ortega-Andrade et al. 2015, Lee et al. 2021). Additionally, species distribution models (SDMs) attempt to estimate geographically in space the potential past, present or future distributions of one or more species (Peterson et al. 2008). ENMs and SDMs respond to different approaches, although in many research studies, both terms have been used synonymously (Peterson and Soberón 2012). For both, the input includes points of occurrence, they can be analysed with the same algorithms and they can have maps as output. ENMs focus mainly on the estimation of the fundamental niche and emphasis on abiotic environmental variables; on the other hand, SDMs concentrate on estimation of environmental space in geographic space and, thus, examine distributions precisely (Peterson and Soberón 2012, Soberón et al. 2017, Melo-Merino et al. 2020).

Thus, considering the lack of information regarding BSF distribution, our study aims to map BSF's current worldwide potential distribution, identify key climatic factors associated with its distribution and predict future potential distribution under a global climate change scenario.

## Material and methods

### Data collection and curation

We collected *H.illucens* occurrence data (Suppl. material 1) from the Global Biodiversity Information Facility website (https://www.gbif.org/) and the iNaturalist platform (https://www.inaturalist.org). The selection of data consisted in manual curating identified records and corroborating them through the minimum visual verification of the following morphological diagnostic characters for this species: eyes bare; frons almost entirely dark brown to black; antennae twice as long as the head; scutellum without spines; darkened; upper fronts with a yellow spot on either side above the middle and another at the vertex; smoky wings; first two hind tarsomeres white or yellow; tibiae in large part black or brownish; abdomen broad, not constricted, with five visible segments; second abdominal segment with a transparent, stained glass area divided centrally by a narrow longitudinal black portion; head, thorax and abdomen blackish (Curran 1941, Navarro and Peris 1991, Lessard et al. 2019, Fachin and Hauser 2022).

**Figure 1.**
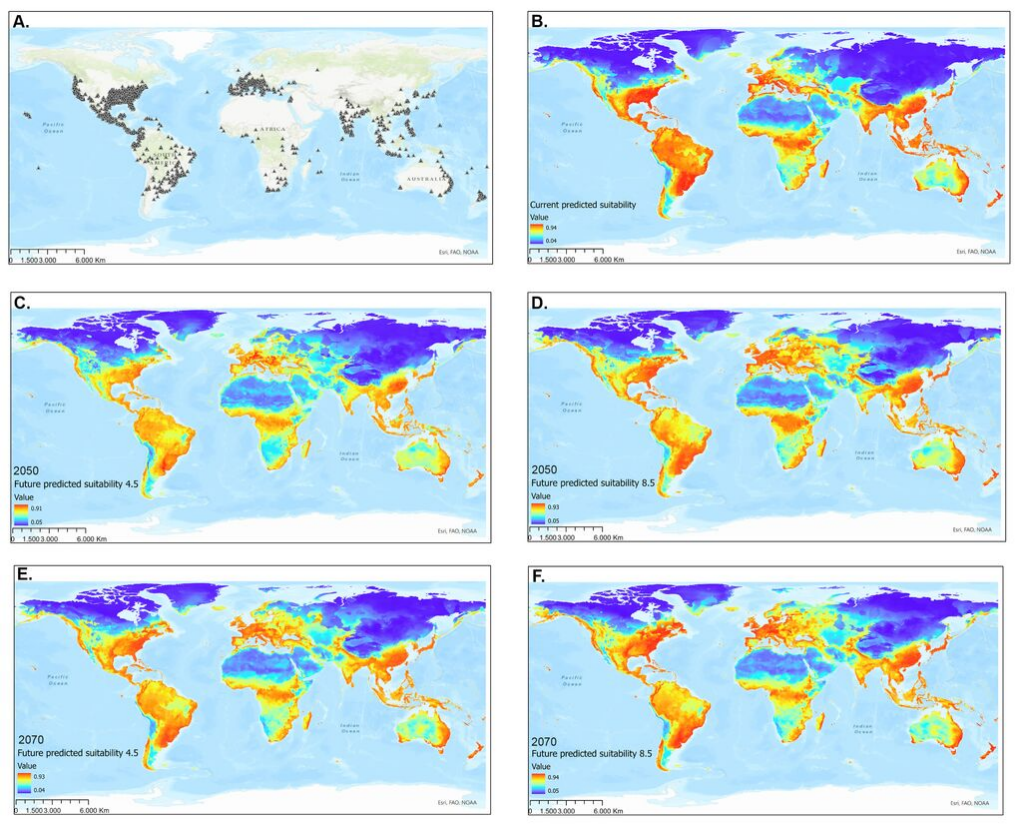
Distribution models of *Hermetiaillucens*. **A** Occurrence records (2,988) from iNaturalist and GBIF; **B** Current global potential distribution **C** Global potential distribution under RCP 4.5 to 2050; **D** Global potential distribution under RCP 8.5 to 2050; **E** Global potential distribution under RCP 4.5 to 2070; **F** Global potential distribution under RCP 8.5 to 2070. The colour gradient scale represents the probabilities of habitat suitability for the distribution of the species, being blue = 0 and red = 1.

Only the records with uploaded images were considered in the current analysis because this is a criterion for verifying the insect's identity through visual identification. We found 6,171 occurrence data for the species in GBIF, gathered from 1897 to 2020 (Global Biodiversity Information Facility, GBIF 2021). In order to avoid temporal biases, we considered the occurrence records made since 1980. Of this total, only 4,002 records met the curation and temporal criteria for model construction (Suppl. material 1). Of these, we finally chose 2,988 independent records (Suppl. material 2) (Fig. 1A), that is, eliminating duplicate or very close occurrences (within 1 кm^2^) to avoid spatial autocorrelation of the data (Sillero et al. 2021) and temporal biases. To unify the coordinate system, we transformed *H.illucens* occurrence records to decimal degrees.

### Environmental Information

WorldClim version 2.1 (http://www.worldclim.org) provided 19 environmental variables to construct an exploratory model of the potential distribution of the species. The variables have an approximate resolution of 1 кm^2^ (Hijmans et al. 2005). To avoid multicollinearity between environmental predictor variables and subsequent instability in the estimation (Dormann et al. 2012), we included the correlation coefficient estimation and the Variance Inflation Factor (VIF). We used an approach that calculates the correlation coefficients of the bioclimatic variables and identifies the pairs of strongly correlated variables. Then, the variable with the highest VIF is removed, the procedure is repeated until there are no correlated variables (Naimi and Araújo 2016). The remaining non-correlated variables were selected through the VIFCOR function in the USDM package for R (Naimi et al. 2014). This function served to calculate the variance inflation factor for excluding variables that are shown to be highly correlated (Naimi et al. 2014).

### Species distribution modelling

Within BAM (i.e. A. environmental conditions; B. biotic interactions; and M. accessible area scheme to generate SDMs) (Soberon and Peterson 2005, Cobos et al. 2019), the accessible area with ideal conditions for the species to occur was considered the entire globe, except for the Arctic and Antarctic Regions. Given that, *H.illucens* is a globally and widely distributed species of economic interest that has colonised various geographic bioregions and part of the objectives of our study is to identify potential areas where the species could occur both in the present and in the future. Therefore, we have considered the aforementioned terrestrial and insular surface as accessible areas (Fig. 1A). Similar approaches for invertebrates have been considered in several studies (Szyniszewska and Tatem 2014, Dos Santos et al. 2017, Reyes and Lira-Noriega 2020).

With the chosen environmental variables, the SDM package (Naimi and Araújo 2016) was used to perform species distribution modelling (SDM) in R 4.1.1 (R Core Team 2021). The package includes several correlative models and generates ensembles of models, avoiding limitations regarding comparisons of multiple SDMs (Naimi and Araújo 2016). Data were fitted to six modelling algorithms: generalised linear model (GLM), support vector machine (SVM), random forests (RF), boosted regression trees (BRT), multivariate adaptive regression spline (MARS) and maximum entropy (MAXENT) (Phillips et al. 2006). We included these algorithms as they have been widely used for the potential reconstruction of the distribution of biodiversity. In addition, they take into account variations related to the nature of the data as the number of records and having a relatively medium to high performance (Konowalik and Nosol 2021).

The algorithms included 30 replicates per method and bootstrap for data partitioning (Suppl. material 3). We included the following time frameworks to be modelled: potential distribution in the present and future distribution in the years 2050 and 2070 under climate change scenarios RCP 4.5 and 8.5, with the circulation model BCC-CSM2-MR derived from Coupled Model Intercomparison Project (CMIP6). We chose this model, based on its improved performance compared to the previous version. This model considers the energy budget at the top of the atmosphere (precipitation, surface air temperature and atmospheric circulation for the global regions); sea surface temperature and climate variations considering the current global warming trend (Wu et al. 2021). RCP 4.5 scenario contemplates the lowest emissions, based on improving technologies to reduce greenhouse effect emissions (Clark et al. 2017). On the other hand, high and constant emissions characterise the RCP 8.5 scenario. It is also constructed upon the assumption that policies to control concentrations of greenhouse gases would not change (Riahi et al. 2011).

### Model Evaluation

Model performance and accuracy evaluation included the following statistics indicators: Receiver Operating Characteristic (ROC) curve with the Area Under the Curve (AUC), partial-area ROC (as the ratio of AUC to the null expectation), Correlation Coefficient (COR) and the True Skill Statistics (TSS). The AUC-ROC and partial-area ROC are suitable to evaluate the model's ability to predict better than chance, considering the genuinely positive and false-positive rates (Hanley and McNeil 1982, Lobo et al. 2008, Peterson et al. 2008). Models with values over 0.8 are considered optimal (Elith et al. 2006). TSS includes omission and commission errors, but is not affected by the size of the validation set (Allouche et al. 2006). COR allows comparisons between models and contributes to understanding AUC and TSS methods (Elith et al. 2006). We did not include the Akaike Information Criterion (AIC) as an evaluation criterion because the information captured by this criterion would not be related to predictive accuracy in geographical distributions (Velasco and González-Salazar 2019). As part of evaluating model performance, the data were split by 25% as a test and 75% as a training fraction (Menéndez-Guerrero and Graham 2013).

Finally, the best candidate models were modelled as an ensemble model. The cut-off threshold for the potential distribution maps of absence and presence was based on the average of all the models of the AUC and TSS indicators; in general, these indicators tend to favour models with a more significant number of occurrences (Allouche et al. 2006). The threshold selected to plot habitat suitability was 0.7638011. It was assumed that habitat suitability meets optimal climatic conditions for establishing the species (Lobo et al. 2008, Kumar et al. 2015, Ortega-Andrade et al. 2015). All the potential distribution prediction maps were developed in ArcGis Pro (Environmental Systems Research Institute (Esri) 2021) and R (R Core Team 2021) the inherent calculations.

## Results

### Variable Correlation

Thirteen out of the nineteen input variables had collinearity problems. The linear correlation coefficients ranged between minimum correlation (bio19 ~ bio8) = 0.1154862 and maximum correlation (bio9 ~ bio8) = 0.6247589. We identified six non-correlated variables. A total of 150 models were built (Suppl. material 4).

**Figure 2.**
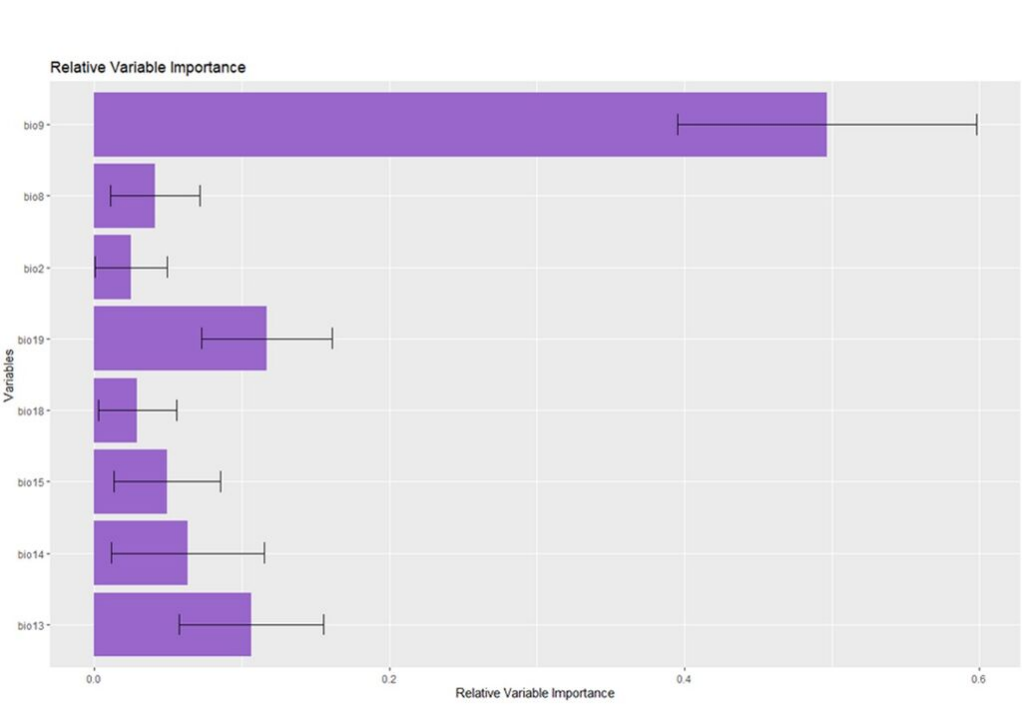
Relative environmental variables importance.

All the methods used demonstrated exemplary performance and predictive power (Table 1). However, GLM was the model with the lowest performance, so it was not used to generate the model ensemble. In this sense, the model was assembled considering the performance of the models SVM, RF, BRT, MARS and Maxent and was evaluated with the partial-area ROC approach. In addition, the mean temperature of the driest annual quarter (Bio 9) contributed significantly to the relative variables' importance (Fig. 2).

### Potential distribution under current climatic conditions

According to the predictions, under current climatic conditions, *H.illucens* is potentially established in much of the sub-humid to humid tropics and subtropics (Fig. 1B). The modelled suitability of the climate for the BSF fits well with the known occurrences of this species in tropical America. The results indicate that the coasts (east, west and south) of Australia, Southeast Asia, tropical Africa, Southeast Africa, the Mediterranean region of Europe, central and the southeast USA offer optimal climatic conditions for establishing the BSF. All oceanic islands between the parallels 50° N and 50° S show habitat suitability greater than 0.8.

The model also predicts that the United States (Texas to Virginia), Mexico, Central America and South America (excluding the dry-lands of south-western Ecuador, Peru, Chile and southern Argentina) are optimal habitats for BSF. Our results suggest that suitability decreases towards the Poles, generating marginal zones in other dry-lands of the world: the Middle East, South Africa and central Australia (Fig. 1B).

**Figure 3.**
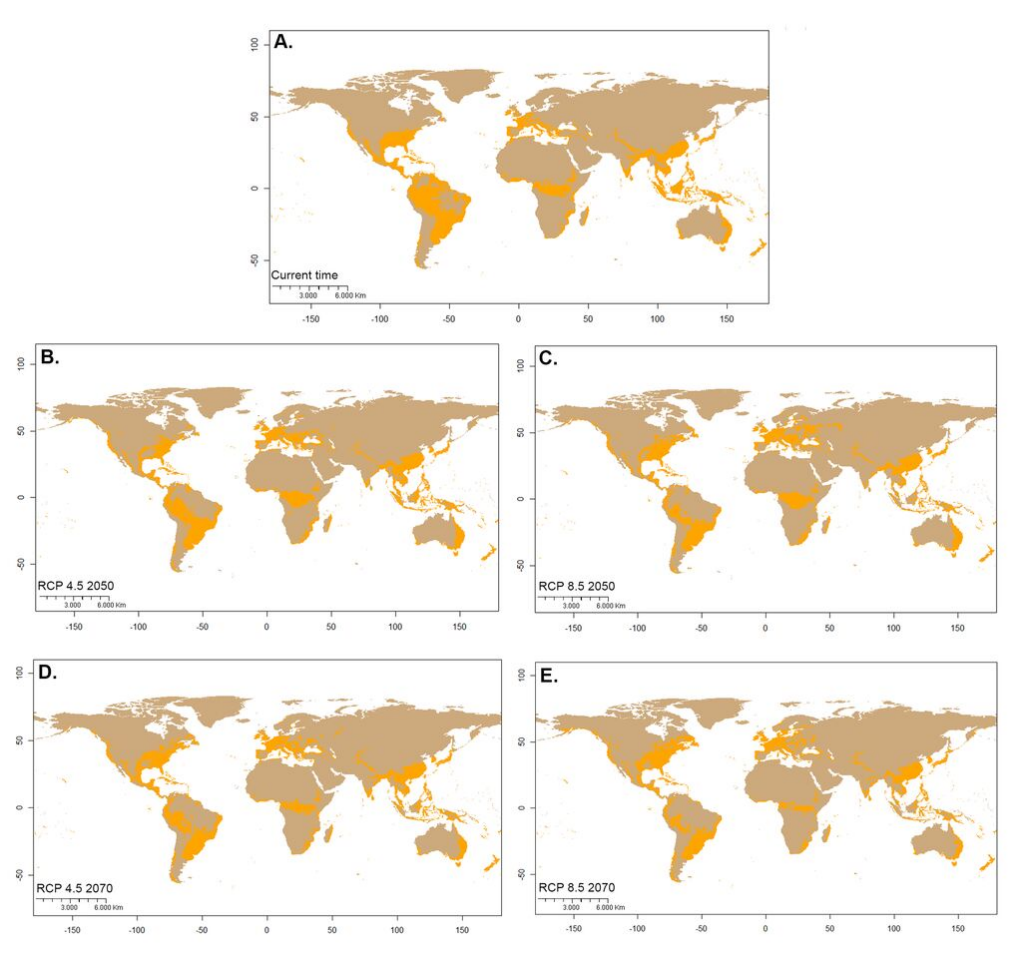
Potential distribution of *Hermetiaillucens* considering a cut-off threshold. Orange represents presence and brown absence. **A** Current potential distribution; **B** RCP 4.5 2050 potential distribution; **C** RCP 8.5 2050 potential distribution; **D** RCP 4.5 2070 potential distribution; **E** RCP 8.5 2070 potential distribution.

**Figure 4.**
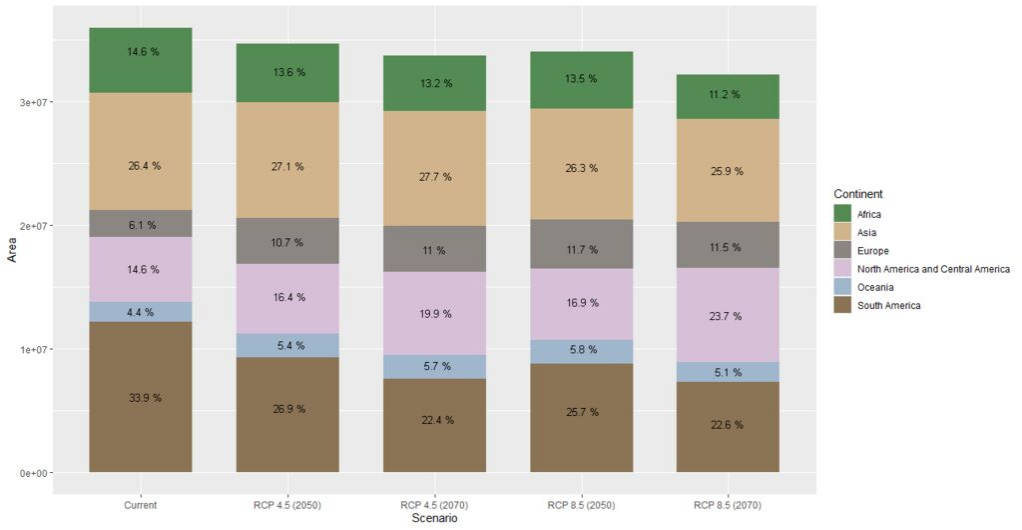
Area of potential distribution of *Hermetiaillucens* in five scenarios.

Using a cut-off threshold with habitat suitability, 40,139,897 km^2^ of the world's land surface is climatically suitable (Fig. 3). By greater than 0.7638011 quantifying the proper surface area for each continent, South America presents the most considerable amount of territory (33.4%), followed by Africa (22.9%) and Asia (17.2%) (Fig. 4).

### Potential distribution of Hermetiaillucens in the future

**Figure 5.**
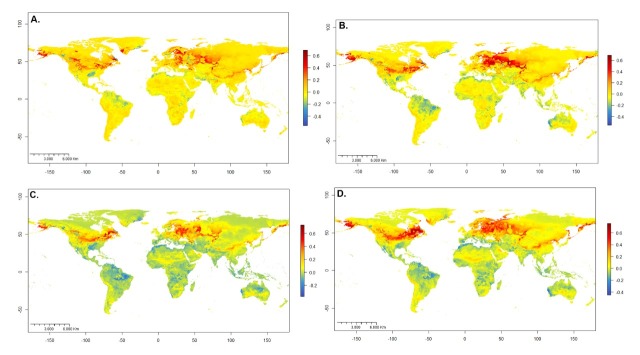
Change between the current and future potential distribution of *Hermetiaillucens*. The red areas represent considerable future changes. **A** changes between current climate and RCP 4.5 2050; **B** changes between current climate and RCP 8.5 2050; **C** changes between current climate and RCP 4.5 2070; **D** changes between current climate and RCP 8.5 2070.

Model performance under different RCPs indicated significant habitat suitability reductions across the species' presence range. A suitable habitat loss gradient is seen as the years and RCP increase. Compared to the current ideal habitat extension, the 4.5 model in 2050 (Fig. 1C) expresses a reduction of 3.6%, followed by the 8.5 model in 2070 (Fig. 1D) with 5.33%, closely followed by the 4.5 scenarios in 2070 (Fig. 1E) with 6.32%. The most drastic change is model 8.5 in 2070 (Fig. 1F), with 10.58% reduction (Figs 4, 5).

**Figure 6.**
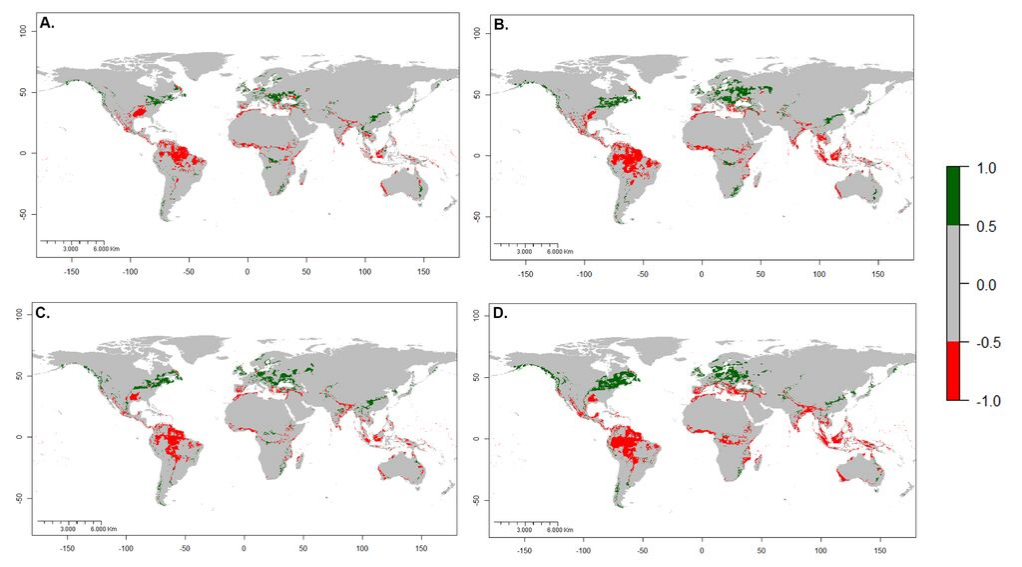
Potential geographic areas where *Hermetiaillucens* would lose (red) or gain (green) suitability for future distribution. **A** RCP 4.5 2050; **B** RCP 8.5 2050; **C** RCP 4.5 2070; **D** RCP 8.5 2070.

The areas most likely to lose BSF suitability in the future are the interior of South America, especially the Amazon Basin, southern USA, central Africa, Mediterranean coasts, Southeast Asia, insular Asia and coasts of Australia. At the same time, the suitability of the habitat would progressively increase in continents with high latitudes, such as North America, Europe and Asia, especially around the 40° N parallel. The same effect seems to manifest below the 40° S parallel in America, Africa and Australia (Figs 5, 6).

**Figure 7.**
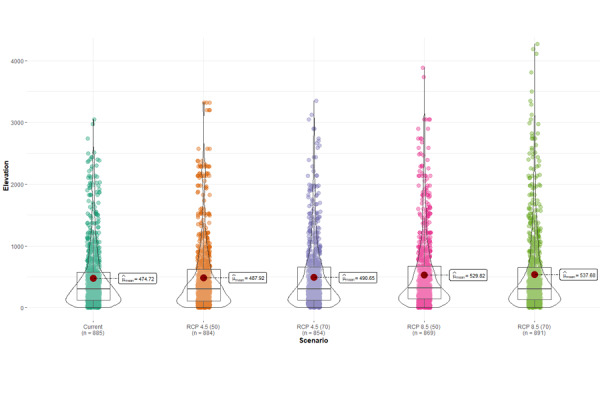
Elevation values (m a.s.l.) considering current climate and different climate change scenarios.

Our models also suggest that BSF will gradually climb in elevation. It is currently known that the species is concentrated from sea level to 2800 m a.s.l. (pers. obs.). According to our projections under the proposed climate scenarios, it could reach up to 4000 m a.s.l. (Fig. 7). However, we understand the limitations of the models and recommend caution in interpreting results.

## Discussion

The suitability of the habitats responds to the climatic variables described in our model. We found that the global distribution of *Hermetiaillucens* is mainly influenced by temperature and precipitation. The *Mean temperature of the driest annual quarter* (Bio9) plays a fundamental role in its dispersion. According to physiological laboratory experiments performed by Tomberlin et al. (2009), the development of BSF at 27°C was delayed by 11% compared to the development at 30°C and, in turn, temperatures higher than 35°C affected the survival of the species. The result could explain the null suitability in cold areas and desert areas or areas with high temperatures in our model.

The second most influential variable was *Precipitation of the coldest annual quarter* (Bio19) and *Precipitation of the wettest month* (bio13), both directly related to humidity. Tomberlin et al. (2009) and Holmes et al. (2012) suggested that the relative environmental humidity influences egg hatching and the success of the emergence of adults. Indeed, Tomberlin et al. (2009) assessed that temperature and humidity are positively correlated with females' oviposition in this species. This statement would explain the wide range of suitability for this species on all continents, mainly in tropical and subtropical areas. The influence of temperature and humidity on the physiology of holometabolic insects is a generalised pattern; small changes in these variables can affect the survival of immature stages, such as eggs and larvae, being factors that determine optimal periods for reproduction (Phillips et al. 2006,Chia et al. 2018).

The current model predicted by this study demonstrates habitat suitability for the BSF in tropical and subtropical areas globally. In this model, BSF presents a marked distribution in the tropical belt of America, Africa, Asia and the Mediterranean coasts. On the other hand, desert areas or low temperatures habitats showed poor suitability for the species. Our model indicates that *H.ilucens* would not be distributed in dry-lands from Peru to Chile, the Andes Mountains in South America, dry-lands of North America, North Africa, the Middle East, Russia and central Australia. Our model matches the collected historical occurrence records (Global Biodiversity Information Facility, GBIF 2019), with most sites presenting at least one occurrence record. On the other hand, this species could be affected by changes in humidity expressed in the drying of its habitat. Indeed, climate change has been shown to accelerate hydrological processes, so drought conditions are more rapidly installed and intense (Mukherjee et al. 2018). Possibly more prolonged drought regimes worldwide, resulting from climate change, could reduce the distribution of *H.illucens*.

Numerous historical records from the late 19^th^ and early 20^th^ centuries document the presence of the species in the New World (Marshall et al. 2015, Kaya et al. 2021). In contrast, the oldest records from the other regions are considerably more recent (Ståhls et al. 2020). In this sense, all Old-World, Australian and Oceanic populations are considered introduced. The chronology of records in Africa, Europe, Asia and Australia indicates relatively recent populations in these non-native habitats. The evidence from the occurrence records matches the molecular evidence-based demographic trajectory (Kaya et al. 2021). Some hypotheses have arisen in this regard, for example, according to Benelli et al. (2014), BSF has presumably been introduced in the Old World about 500 years ago during the European invasion of America. Based on literature records, the species is now widely distributed worldwide (Woodley 2001).Woodley 2001 Several authors acknowledge that its origin is Neotropical, extending from northern South America to the south-western United States. Consequently, it has spread to other biogeographic regions, possibly by human action (Leclerq 1962, Navarro and Peris 1991, Roháček and Hora 2013, Marshall et al. 2015).

Consistent records of this species in new sites, mainly in the Palaearctic, are found along coasts and islands, suggesting that maritime transport may play a role in possible repeated accidental introductions (Gladun 2019, Ståhls et al. 2020). The same situation occurs in south-eastern Asia, where the entry of BSF seems to be closely related to World War II and the massive amount of material and troops that were shipped between islands (M. Hauser, personal communication). Focusing on these common antecedents, our model showed high suitability (> 0.8) on tropical coasts, but especially on all oceanic islands between the parallels 50° N and 50° S. Species with substantial distributions often consist of complexes of species or several different evolutionary units. Notwithstanding, molecular analyses, performed on diverse populations of *H.illucens* established worldwide, indicate that it is a single species that exhibits genetic diversity extremely distinguished, but simultaneously with good reproductive compatibility (Ståhls et al. 2020).

The BSF has been classified as hemi-synanthropic; it lives around human populations in semi-rural and rural landscapes (Greenberg 1971). Its ability to use a wide range of decomposing organic matter has allowed it to penetrate new and remote regions.However, its spread may have been accelerated in thhe first instance by the mobilisation of ships for trade, conquest or warfare between countries. (Newton et al. 1977, Craig Sheppard et al. 1994, Marshall et al. 2015). Furthermore, BSF appears to spread accidentally by trading waste and products (Roháček and Hora 2013). So far, there are no known negative impacts due to the introduction of BSF in new environments. However, it is necessary to deepen the knowledge of how it competes for resources with certain native species of Stratiomyidae in Asia, Africa or Australia. Therefore, with the existing information, it cannot be categorised as an invasive species (Pagad et al. 2015). On the contrary, this species re-introduces nutrients by converting decaying organic matter into protein (Craig Sheppard et al. 1994, Müller et al. 2017).

Based on a future scenario in which climate change would modify the planet's climatic conditions, the RCP 4.5 and 8.5 scenarios have been taken as reference in 2050 and 2070 (Riahi et al. 2011). Our model predicts that *H.illucens* could progressively lose up to 10% of suitable habitats by 2070. Expansions of the species' range to high latitudes, especially north of parallel 40° N in America and Europe, indicate that a temperature threshold currently limits BSF distribution. However, the model suggests that it would exceed this threshold in any of the scenarios we modelled. This agrees with the projections for pest insects in the plantations of the Scandinavian Peninsula. Hof and Svahlin (2016) predicted that the distribution of pest species would expand in 2070, especially towards regions with higher elevation and towards the geographic north.

According to similar models, habitat suitability in South America, Africa, Southeast Asia and Australia would decrease due to increased temperature stress. Similar results were reported for other dipteran crop pests, such as *Bactroceradorsalis* (Hendel) (Stephens et al. 2007) and *Anastrephaobliqua* (Macquart) (Fu et al. 2014). Furthermore, climatic changes displaced other species, such as sandflies (Psychodidae) (Moo-Llanes et al. 2013) and the invasive species of the tropical fire ant, *Solenopsisgeminata* (Fabricius) (Hymenoptera, Formicidae) (Lee et al. 2021). Therefore, environmental modifications caused by climate change, particularly changes in rainfall and temperature patterns, would influence the survival capacity of insects by affecting their reproductive performance, the survival of their larvae and symbiotic or mutualistic interactions with other organisms (Mellanby et al. 1967, Hurlbert and Liang 2012, Gao and Shi 2021).

Our model predicts that the current altitude range of *H.illucens* would change under climate change scenarios. It is projected that this species could colonise elevations 2800 m, higher than what is currently recorded. This coincides with the projections of coleopterans in the mountains of south-eastern Brazil (Colares et al. 2021). For example, Colares et al. (2021) concluded that species of Eumolpinae (Chrysomelidae), under the RCP 4.5 scenario at the end of the century, would have an upward displacement of 460 m in its distribution. However, the introduction of BSF in a new altitudinal range can lead to other consequences, such as changes in the interactions of the species, abundance, phenology, life cycle, local extinction and evolutionary responses in that ecosystem (Menéndez 2007, Sheldon 2019).

Data on the occurrence of *H.illucens* were collected during the confinement of COVID-19, a condition that prevented access to biological collections. The restrictions motivated the use of the world's biodiversity registry repositories, such as the GBIF and iNaturalist platforms. The data provided by the citizen-science observations of the iNaturalist platform served as the primary tool to create the presented models. The morphological characters of the species acted as a delimiting parameter for the selection of data occurrence, mainly due to characteristics visually recognisable by photographs, which showed a pattern in the records throughout its distribution (Ståhls et al. 2020). There are few examples of insect species with easily recognisable characteristics, except for butterflies (Prudic et al. 2017, Prudic et al. 2018). In this sense, the present study shows that wildlife observations in citizen-science projects can accurately predict species distributions when complemented by verification given by specialist researchers working on identifications from the thousands of observations uploaded on platforms, such as iNaturalist (Tiago et al. 2017).

## Conclusions

The present study modelled the potential distribution of *H.illucens* on a global scale in current and future scenarios. The methodology applied six species distribution modelling algorithms, based on bioclimatic variables. The model exhibited a high prediction performance where the mean temperature of the driest annual quarter and precipitation were the key factors influencing the potential distribution of BSF. According to our model, the possible ranges of *H.illucens* would decrease by up to 10.5% in the future due to global warming. The distribution of favourable habitats would change to high latitudes and high elevations.

On the other hand, much of the current optimal habitats would become uninhabitable for the species, mainly at low latitudes. This study provides the distribution of BSF to facilitate its location, management and sustainable use in current and future scenarios. Nevertheless, the model presents a strong warning about global warming consequences, which, in addition to the ecological distribution, threatens the growing production of *H.illucens* as an alternative for waste management. However, the model did not include drought and precipitation indices resulting from climate change, which could reduce *H.illucens* distribution (Mukherjee et al. 2018). It would be advisable for future potential distribution models to include these indices to increase accuracy.

Finally, we should mention that the citizen-science data served to create our models under strict verification and curation filters (Dickinson et al. 2010). This study joins a growing number of studies on the effects of climate change on the distribution of species that use citizen science as a fundamental tool for obtaining primary data (Hurlbert and Liang 2012, Abolafya et al. 2013, Menchetti et al. 2019).

## Supplementary Material

7EE7C54A-9136-565D-BE1F-943D1152F476 10.3897/BDJ.10.e90146.suppl1 Supplementary material 1 Appendix S1.1 Data type *Hermetiaillucens* occurrences Brief description The supplementary material includes *Hermetiaillucens* occurrences collected from the Global Biodiversity Information Facility (GBIF) complemented with iNaturalist. File: oo_754194.csv https://binary.pensoft.net/file/754194 Pazmiño-Palomino Alex and Reyes-Puig Carolina

99DA15C1-8E3B-5759-86DE-4778C0A4AC41 10.3897/BDJ.10.e90146.suppl2 Supplementary material 2 Appendix S1.2 Data type *Hermetiaillucens* occurrences Brief description Supplementary material includes manually curated records from Supplementary material 1.1. File: oo_712779.csv https://binary.pensoft.net/file/712779 Pazmiño-Palomino Alex and Reyes-Puig Carolina

DF0E606D-6B0D-5339-8F76-1144F7569872 10.3897/BDJ.10.e90146.suppl3 Supplementary material 3 Appendix S2 Data type models Brief description Thirty predictions of current potential distribution for each algorithm. File: oo_696524.pdf https://binary.pensoft.net/file/696524 Reyes-Puig Carolina

D958857C-2ACA-52D3-B414-7946CF70DD02 10.3897/BDJ.10.e90146.suppl4 Supplementary material 4 Appendix S3 Data type DM script for *Hermetia* portential distribution Brief description R script used to build the models. File: oo_696494.pdf https://binary.pensoft.net/file/696494 Reyes-Puig Carolina

## Figures and Tables

**Table 1.** Performance evaluation of the six algorithms used to develop the model through the different statistical indicators.

**Methods**	**AUC**	**AUC ratio**	**COR**	**TSS**
**GLM**	0.78	1.52	0.58	0.48
**SVM**	0.92	1.66	0.77	0.74
**RF**	0.99	1.78	0.89	0.88
**BRT**	0.9	1.62	0.72	0.66
**MARS**	0.9	1.65	0.73	0.67
**Maxent**	0.9	1.71	0.72	0.66
