# Supplementary material for: How could climate change influence the distribution of the black soldier fly, Hermetiaillucens (Linnaeus) (Diptera, Stratiomyidae)?
Source: Biodivers Data J. 2022 Oct 17;10:e90146. doi: 10.3897/BDJ.10.e90146 (PMC9836546; doi:10.3897/BDJ.10.e90146)

Supplementary material II. Thirty predictions of current potential distribution for each algorithm

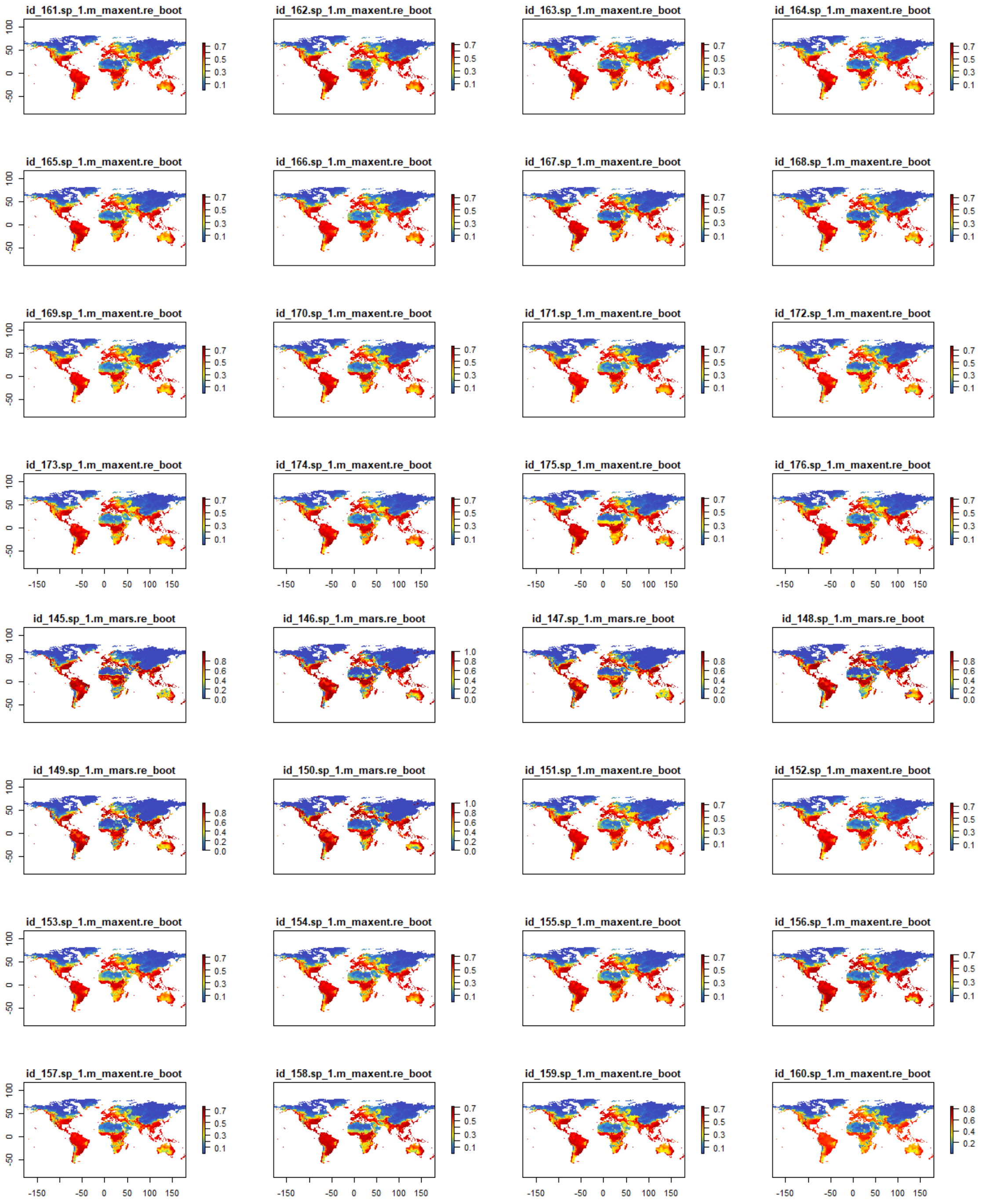

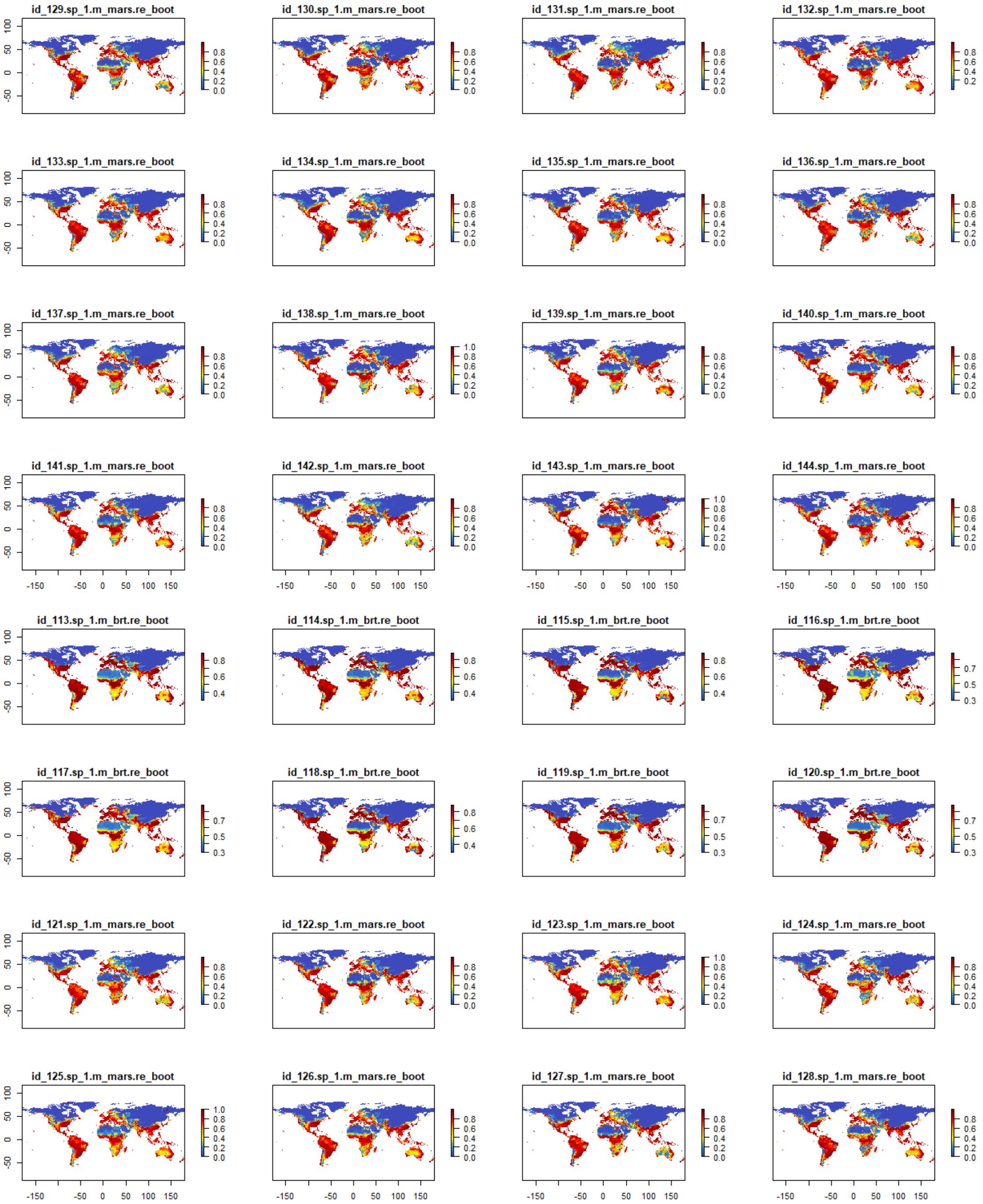
id_129.sp_1.m_mars.re_boot
id_130.sp_1.m_mars.re_boot
id_131.sp_1.m_mars.re_boot
id_132.sp_1.m_mars.re_boot
id_133.sp_1.m_mars.re_boot
id_134.sp_1.m_mars.re_boot
id_135.sp_1.m_mars.re_boot
id_136.sp_1.m_mars.re_boot
id_137.sp_1.m_mars.re_boot
id_138.sp_1.m_mars.re_boot
id_139.sp_1.m_mars.re_boot
id_140.sp_1.m_mars.re_boot
id_141.sp_1.m_mars.re_boot
id_142.sp_1.m_mars.re_boot
id_143.sp_1.m_mars.re_boot
id_144.sp_1.m_mars.re_boot
id_113.sp_1.m_brt.re_boot
id_114.sp_1.m_brt.re_boot
id_115.sp_1.m_brt.re_boot
id_116.sp_1.m_brt.re_boot
id_117.sp_1.m_brt.re_boot
id_118.sp_1.m_brt.re_boot
id_119.sp_1.m_brt.re_boot
id_120.sp_1.m_brt.re_boot
id_121.sp_1.m_mars.re_boot
id_122.sp_1.m_mars.re_boot
id_123.sp_1.m_mars.re_boot
id_124.sp_1.m_mars.re_boot
id_125.sp_1.m_mars.re_boot
id_126.sp_1.m_mars.re_boot
id_127.sp_1.m_mars.re_boot
id_128.sp_1.m_mars.re_boot

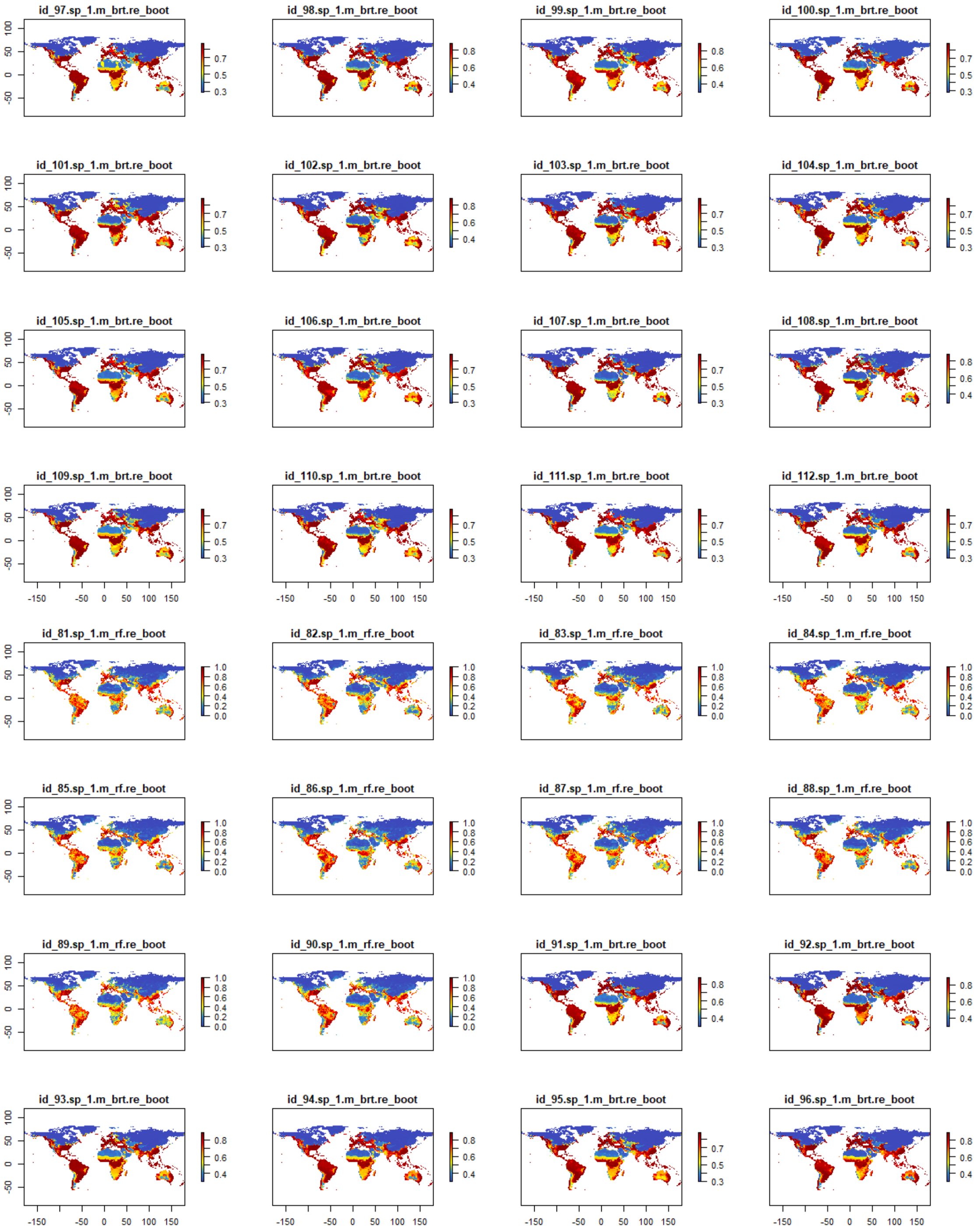
id_97.sp_1.m_brt.re_boot
id_98.sp_1.m_brt.re_boot
id_99.sp_1.m_brt.re_boot
id_100.sp_1.m_brt.re_boot
id_101.sp_1.m_brt.re_boot
id_102.sp_1.m_brt.re_boot
id_103.sp_1.m_brt.re_boot
id_104.sp_1.m_brt.re_boot
id_105.sp_1.m_brt.re_boot
id_106.sp_1.m_brt.re_boot
id_107.sp_1.m_brt.re_boot
id_108.sp_1.m_brt.re_boot
id_109.sp_1.m_brt.re_boot
id_110.sp_1.m_brt.re_boot
id_111.sp_1.m_brt.re_boot
id_112.sp_1.m_brt.re_boot
id_81.sp_1.m_rf.re_boot
id_82.sp_1.m_rf.re_boot
id_83.sp_1.m_rf.re_boot
id_84.sp_1.m_rf.re_boot
id_85.sp_1.m_rf.re_boot
id_86.sp_1.m_rf.re_boot
id_87.sp_1.m_rf.re_boot
id_88.sp_1.m_rf.re_boot
id_89.sp_1.m_rf.re_boot
id_90.sp_1.m_rf.re_boot
id_91.sp_1.m_brt.re_boot
id_92.sp_1.m_brt.re_boot
id_93.sp_1.m_brt.re_boot
id_94.sp_1.m_brt.re_boot
id_95.sp_1.m_brt.re_boot
id_96.sp_1.m_brt.re_boot

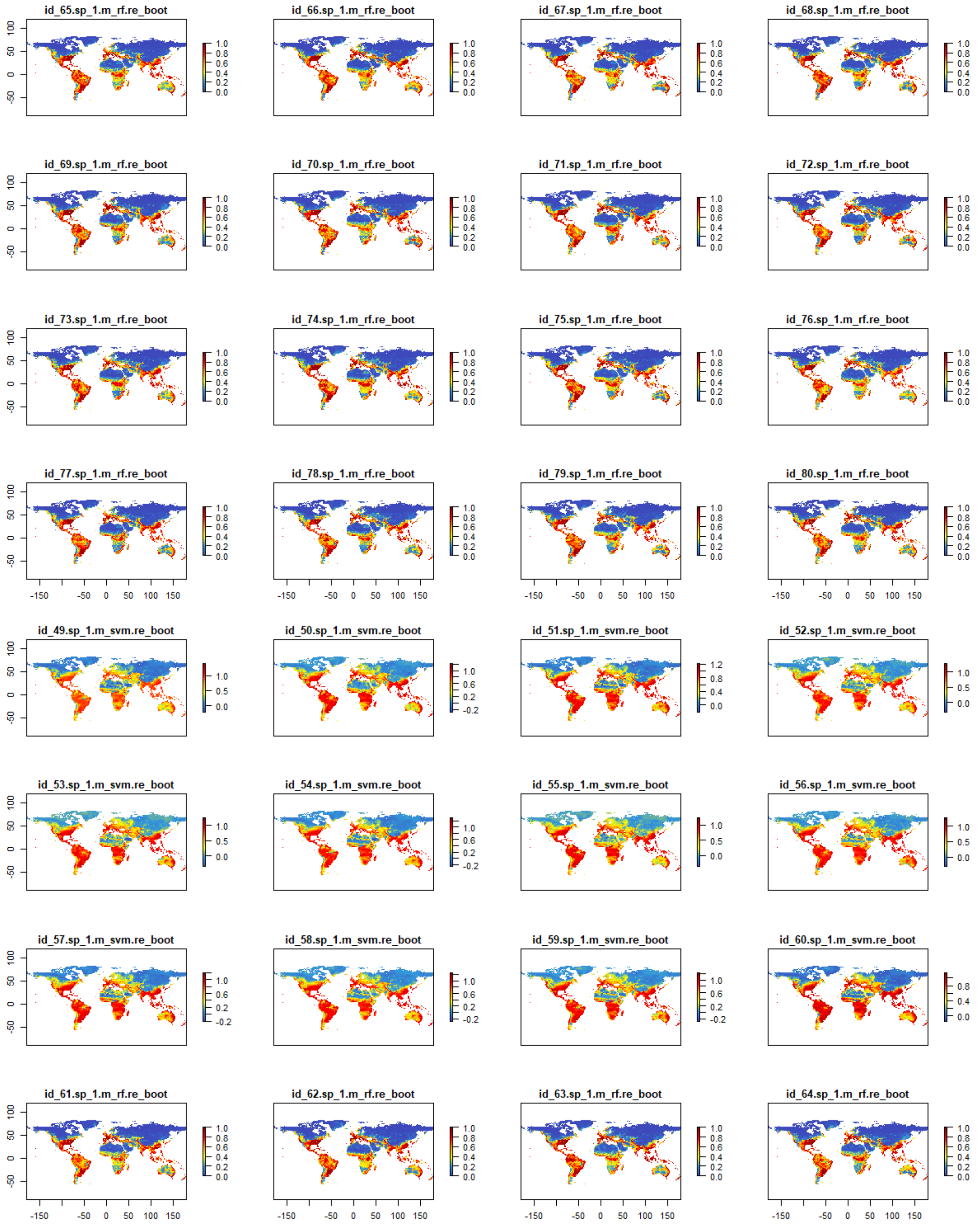
id_65.sp_1.m_rf.re_boot
id_66.sp_1.m_rf.re_boot
id_67.sp_1.m_rf.re_boot
id_68.sp_1.m_rf.re_boot
id_69.sp_1.m_rf.re_boot
id_70.sp_1.m_rf.re_boot
id_71.sp_1.m_rf.re_boot
id_72.sp_1.m_rf.re_boot
id_73.sp_1.m_rf.re_boot
id_74.sp_1.m_rf.re_boot
id_75.sp_1.m_rf.re_boot
id_76.sp_1.m_rf.re_boot
id_77.sp_1.m_rf.re_boot
id_78.sp_1.m_rf.re_boot
id_79.sp_1.m_rf.re_boot
id_80.sp_1.m_rf.re_boot
id_49.sp_1.m_svm.re_boot
id_50.sp_1.m_svm.re_boot
id_51.sp_1.m_svm.re_boot
id_52.sp_1.m_svm.re_boot
id_53.sp_1.m_svm.re_boot
id_54.sp_1.m_svm.re_boot
id_55.sp_1.m_svm.re_boot
id_56.sp_1.m_svm.re_boot
id_57.sp_1.m_svm.re_boot
id_58.sp_1.m_svm.re_boot
id_59.sp_1.m_svm.re_boot
id_60.sp_1.m_svm.re_boot
id_61.sp_1.m_rf.re_boot
id_62.sp_1.m_rf.re_boot
id_63.sp_1.m_rf.re_boot
id_64.sp_1.m_rf.re_boot

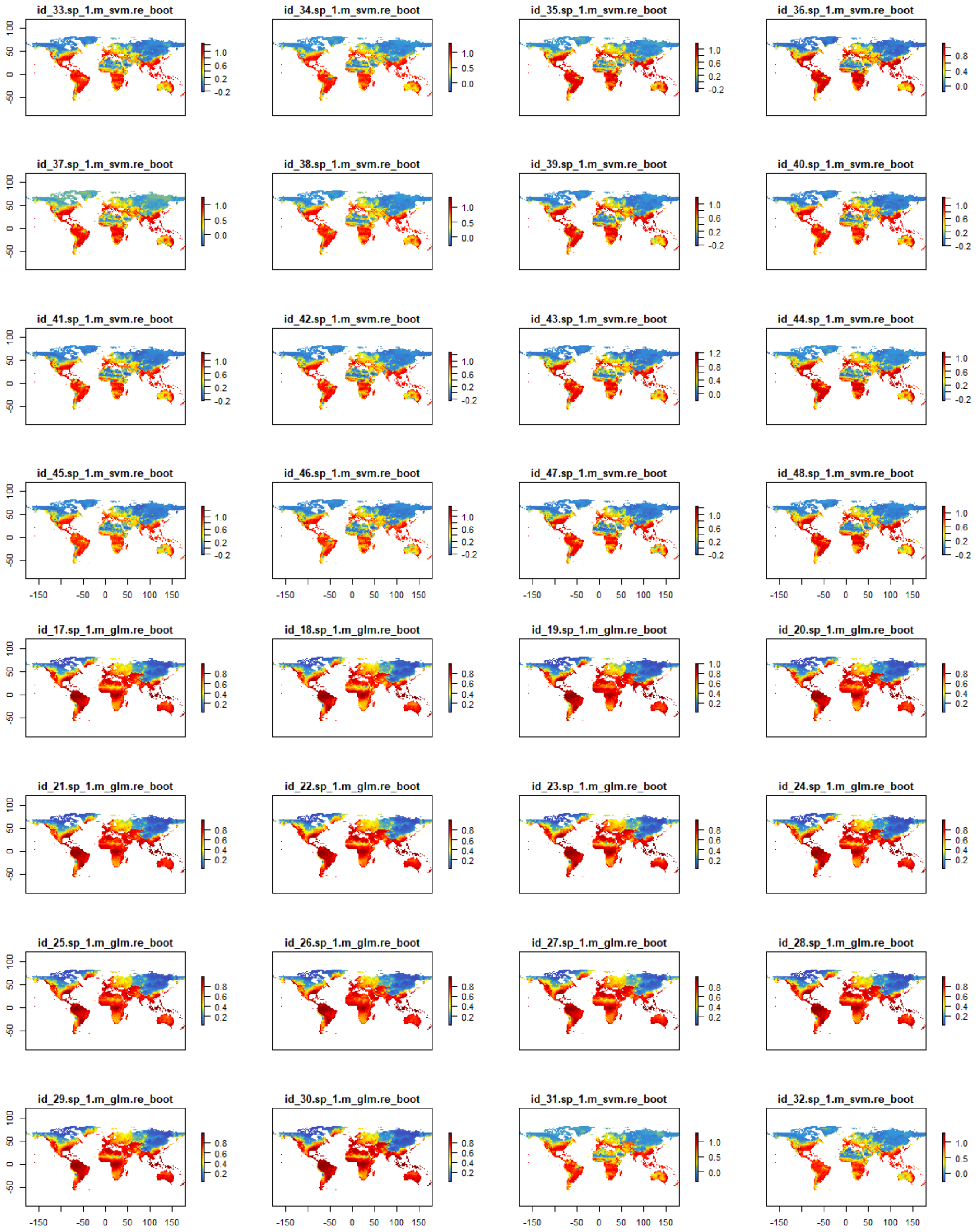
id_33.sp_1.m_svm.re_boot
id_34.sp_1.m_svm.re_boot
id_35.sp_1.m_svm.re_boot
id_36.sp_1.m_svm.re_boot
id_37.sp_1.m_svm.re_boot
id_38.sp_1.m_svm.re_boot
id_39.sp_1.m_svm.re_boot
id_40.sp_1.m_svm.re_boot
id_41.sp_1.m_svm.re_boot
id_42.sp_1.m_svm.re_boot
id_43.sp_1.m_svm.re_boot
id_44.sp_1.m_svm.re_boot
id_45.sp_1.m_svm.re_boot
id_46.sp_1.m_svm.re_boot
id_47.sp_1.m_svm.re_boot
id_48.sp_1.m_svm.re_boot
id_17.sp_1.m_glm.re_boot
id_18.sp_1.m_glm.re_boot
id_19.sp_1.m_glm.re_boot
id_20.sp_1.m_glm.re_boot
id_21.sp_1.m_glm.re_boot
id_22.sp_1.m_glm.re_boot
id_23.sp_1.m_glm.re_boot
id_24.sp_1.m_glm.re_boot
id_25.sp_1.m_glm.re_boot
id_26.sp_1.m_glm.re_boot
id_27.sp_1.m_glm.re_boot
id_28.sp_1.m_glm.re_boot
id_29.sp_1.m_glm.re_boot
id_30.sp_1.m_glm.re_boot
id_31.sp_1.m_svm.re_boot
id_32.sp_1.m_svm.re_boot

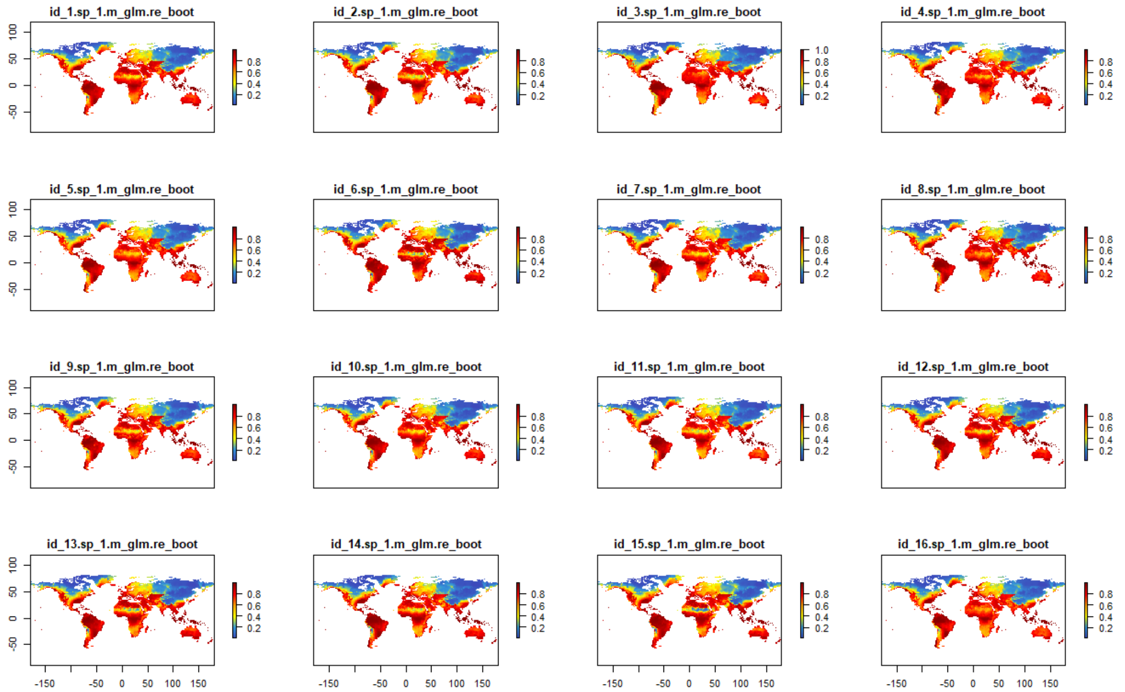
id_1.sp_1.m_glm.re_boot
id_2.sp_1.m_glm.re_boot
id_3.sp_1.m_glm.re_boot
id_4.sp_1.m_glm.re_boot
id_5.sp_1.m_glm.re_boot
id_6.sp_1.m_glm.re_boot
id_7.sp_1.m_glm.re_boot
id_8.sp_1.m_glm.re_boot
id_9.sp_1.m_glm.re_boot
id_10.sp_1.m_glm.re_boot
id_11.sp_1.m_glm.re_boot
id_12.sp_1.m_glm.re_boot
id_13.sp_1.m_glm.re_boot
id_14.sp_1.m_glm.re_boot
id_15.sp_1.m_glm.re_boot
id_16.sp_1.m_glm.re_boot

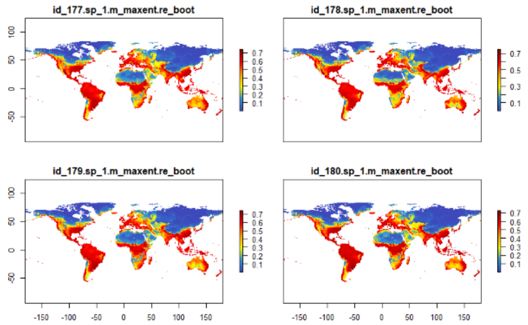
id_177.sp_1.m_maxent.re_boot
id_178.sp_1.m_maxent.re_boot
id_179.sp_1.m_maxent.re_boot
id_180.sp_1.m_maxent.re_boot

Supplementary material I.2. Thirty predictions of potential distribution for RCP 4.5 year 2050 for each algorithm

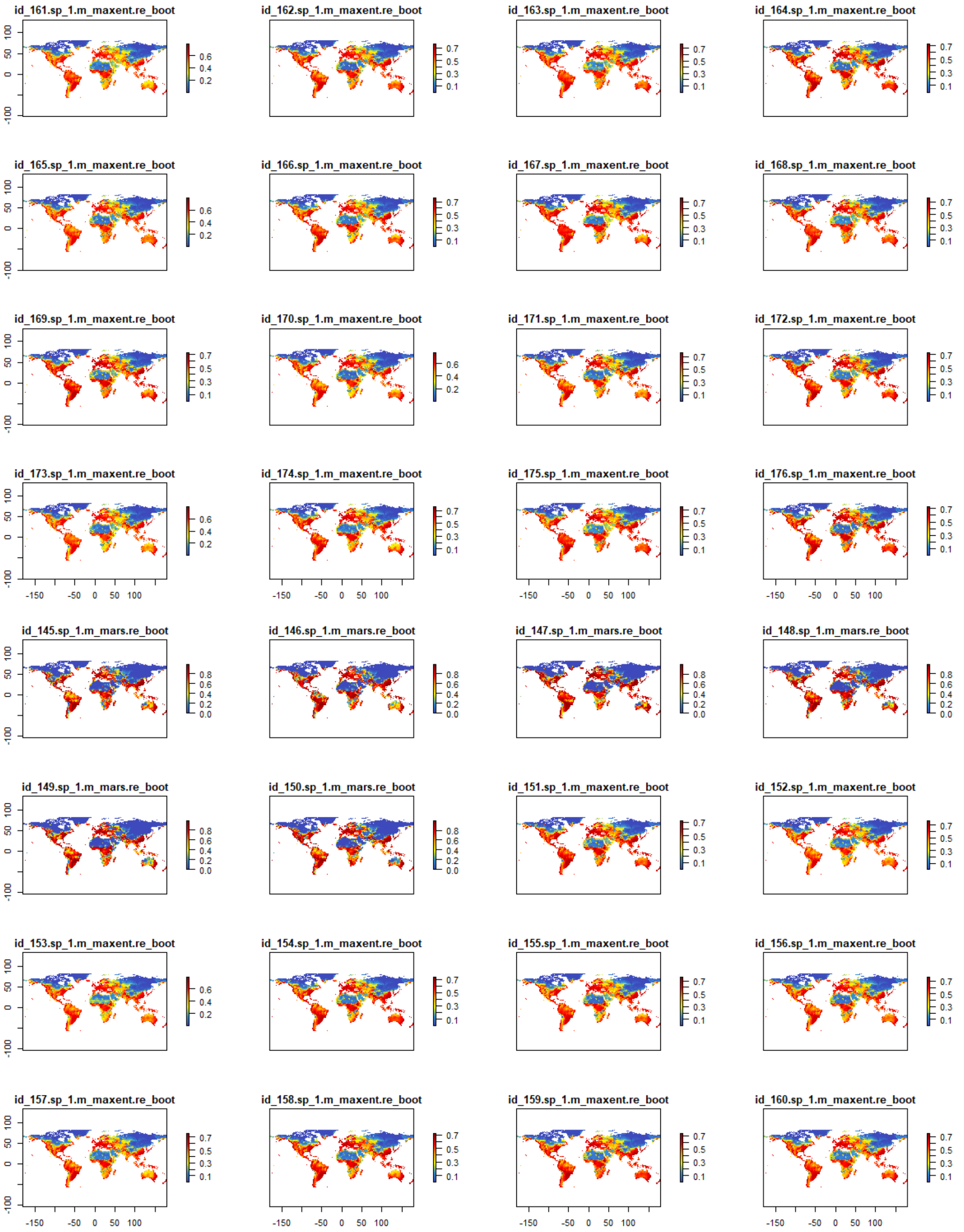

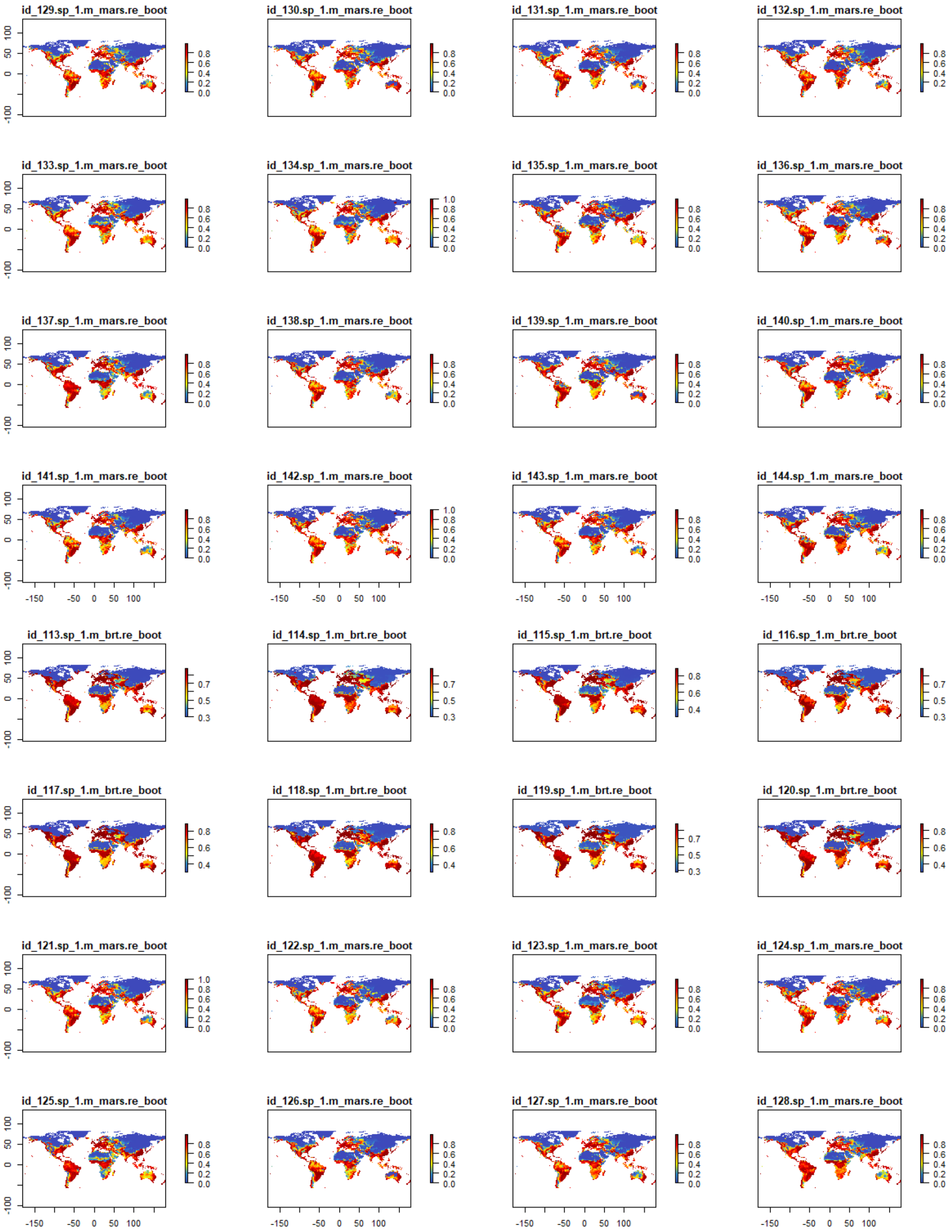
id_129.sp_1.m_mars.re_boot
id_130.sp_1.m_mars.re_boot
id_131.sp_1.m_mars.re_boot
id_132.sp_1.m_mars.re_boot
id_133.sp_1.m_mars.re_boot
id_134.sp_1.m_mars.re_boot
id_135.sp_1.m_mars.re_boot
id_136.sp_1.m_mars.re_boot
id_137.sp_1.m_mars.re_boot
id_138.sp_1.m_mars.re_boot
id_139.sp_1.m_mars.re_boot
id_140.sp_1.m_mars.re_boot
id_141.sp_1.m_mars.re_boot
id_142.sp_1.m_mars.re_boot
id_143.sp_1.m_mars.re_boot
id_144.sp_1.m_mars.re_boot
id_113.sp_1.m_brt.re_boot
id_114.sp_1.m_brt.re_boot
id_115.sp_1.m_brt.re_boot
id_116.sp_1.m_brt.re_boot
id_117.sp_1.m_brt.re_boot
id_118.sp_1.m_brt.re_boot
id_119.sp_1.m_brt.re_boot
id_120.sp_1.m_brt.re_boot
id_121.sp_1.m_mars.re_boot
id_122.sp_1.m_mars.re_boot
id_123.sp_1.m_mars.re_boot
id_124.sp_1.m_mars.re_boot
id_125.sp_1.m_mars.re_boot
id_126.sp_1.m_mars.re_boot
id_127.sp_1.m_mars.re_boot
id_128.sp_1.m_mars.re_boot

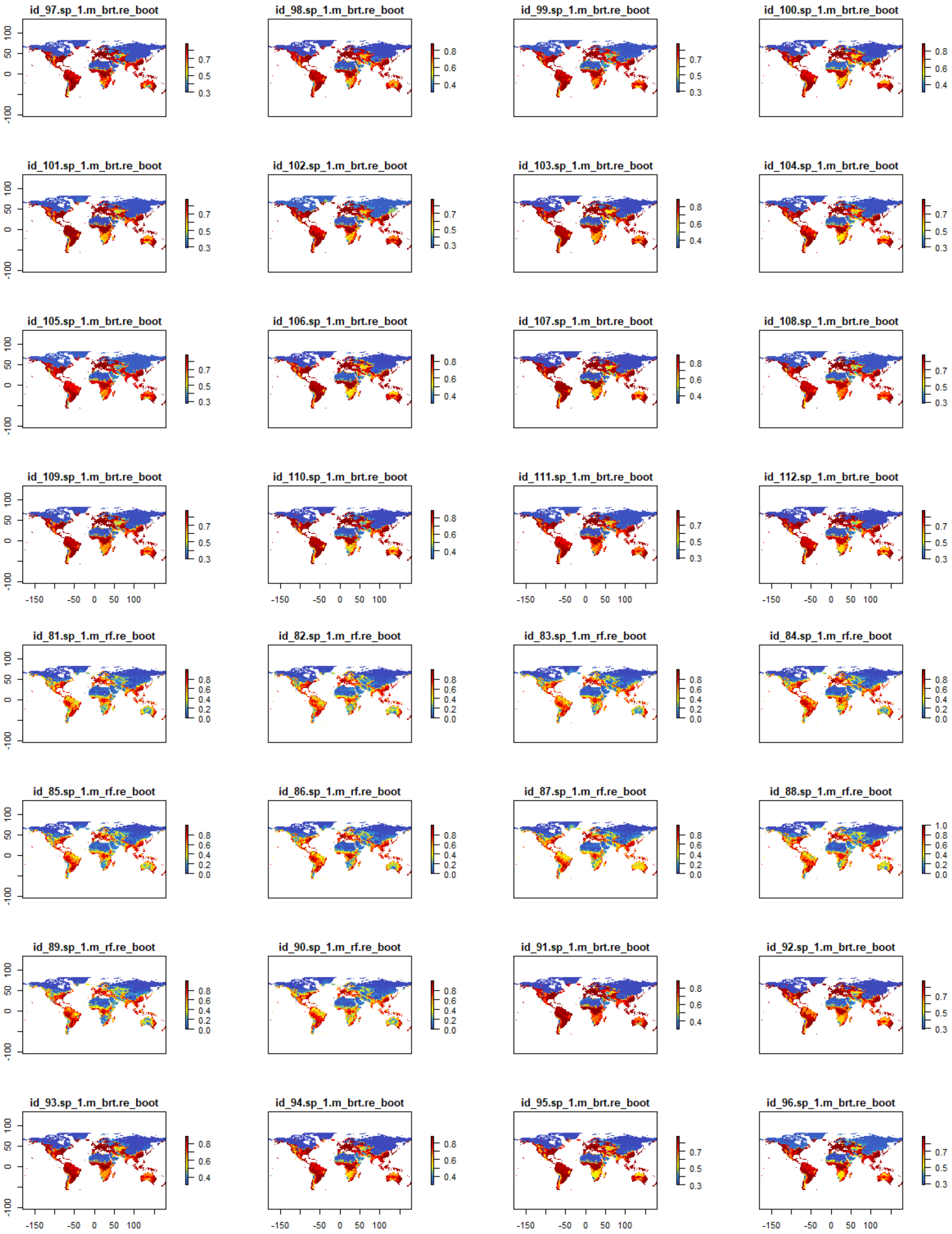
id_97.sp_1.m_brt.re_boot
id_98.sp_1.m_brt.re_boot
id_99.sp_1.m_brt.re_boot
id_100.sp_1.m_brt.re_boot
id_101.sp_1.m_brt.re_boot
id_102.sp_1.m_brt.re_boot
id_103.sp_1.m_brt.re_boot
id_104.sp_1.m_brt.re_boot
id_105.sp_1.m_brt.re_boot
id_106.sp_1.m_brt.re_boot
id_107.sp_1.m_brt.re_boot
id_108.sp_1.m_brt.re_boot
id_109.sp_1.m_brt.re_boot
id_110.sp_1.m_brt.re_boot
id_111.sp_1.m_brt.re_boot
id_112.sp_1.m_brt.re_boot
id_81.sp_1.m_rf.re_boot
id_82.sp_1.m_rf.re_boot
id_83.sp_1.m_rf.re_boot
id_84.sp_1.m_rf.re_boot
id_85.sp_1.m_rf.re_boot
id_86.sp_1.m_rf.re_boot
id_87.sp_1.m_rf.re_boot
id_88.sp_1.m_rf.re_boot
id_89.sp_1.m_rf.re_boot
id_90.sp_1.m_rf.re_boot
id_91.sp_1.m_brt.re_boot
id_92.sp_1.m_brt.re_boot
id_93.sp_1.m_brt.re_boot
id_94.sp_1.m_brt.re_boot
id_95.sp_1.m_brt.re_boot
id_96.sp_1.m_brt.re_boot

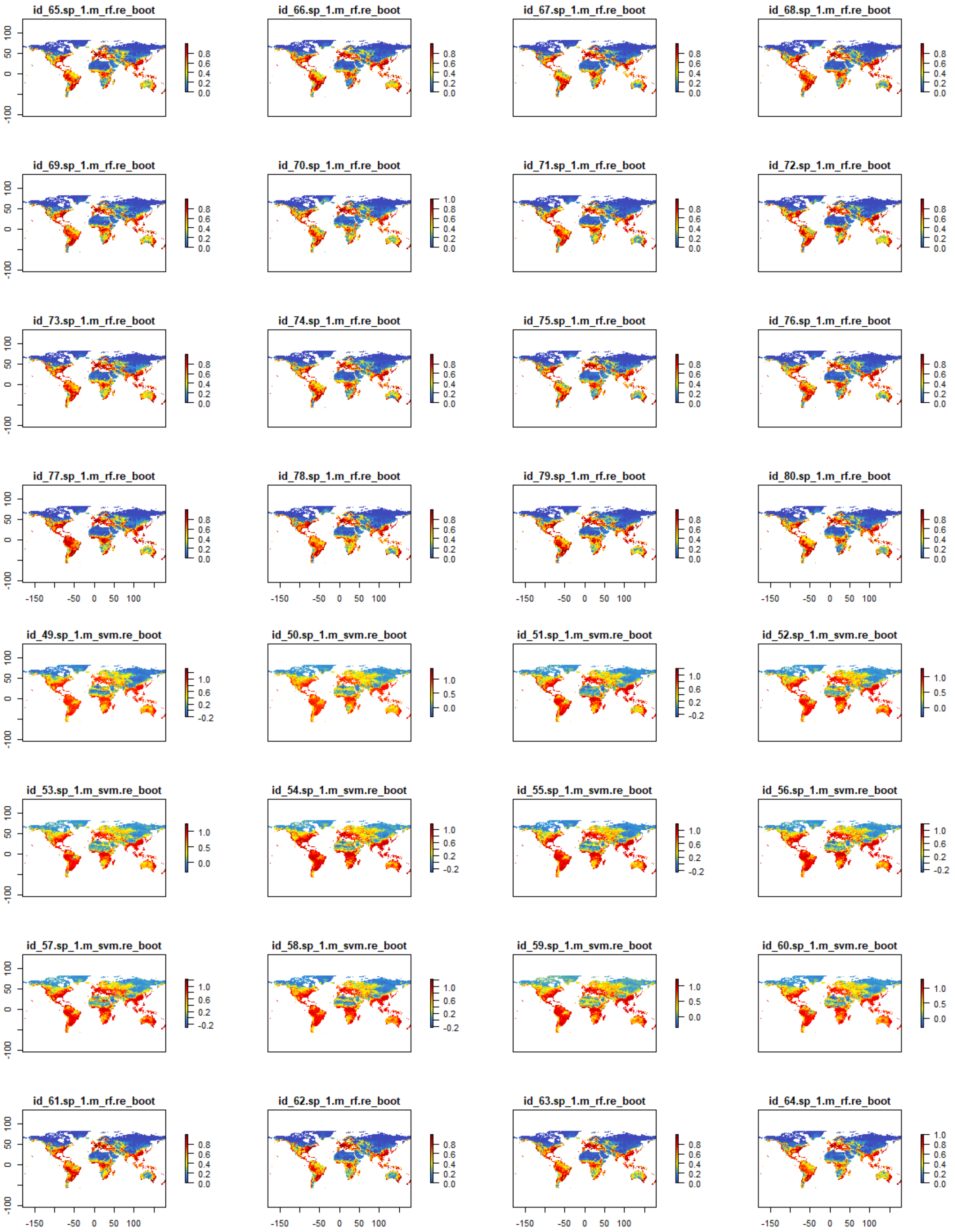
id_65.sp_1.m_rf.re_boot
id_66.sp_1.m_rf.re_boot
id_67.sp_1.m_rf.re_boot
id_68.sp_1.m_rf.re_boot
id_69.sp_1.m_rf.re_boot
id_70.sp_1.m_rf.re_boot
id_71.sp_1.m_rf.re_boot
id_72.sp_1.m_rf.re_boot
id_73.sp_1.m_rf.re_boot
id_74.sp_1.m_rf.re_boot
id_75.sp_1.m_rf.re_boot
id_76.sp_1.m_rf.re_boot
id_77.sp_1.m_rf.re_boot
id_78.sp_1.m_rf.re_boot
id_79.sp_1.m_rf.re_boot
id_80.sp_1.m_rf.re_boot
id_49.sp_1.m_svm.re_boot
id_50.sp_1.m_svm.re_boot
id_51.sp_1.m_svm.re_boot
id_52.sp_1.m_svm.re_boot
id_53.sp_1.m_svm.re_boot
id_54.sp_1.m_svm.re_boot
id_55.sp_1.m_svm.re_boot
id_56.sp_1.m_svm.re_boot
id_57.sp_1.m_svm.re_boot
id_58.sp_1.m_svm.re_boot
id_59.sp_1.m_svm.re_boot
id_60.sp_1.m_svm.re_boot
id_61.sp_1.m_rf.re_boot
id_62.sp_1.m_rf.re_boot
id_63.sp_1.m_rf.re_boot
id_64.sp_1.m_rf.re_boot

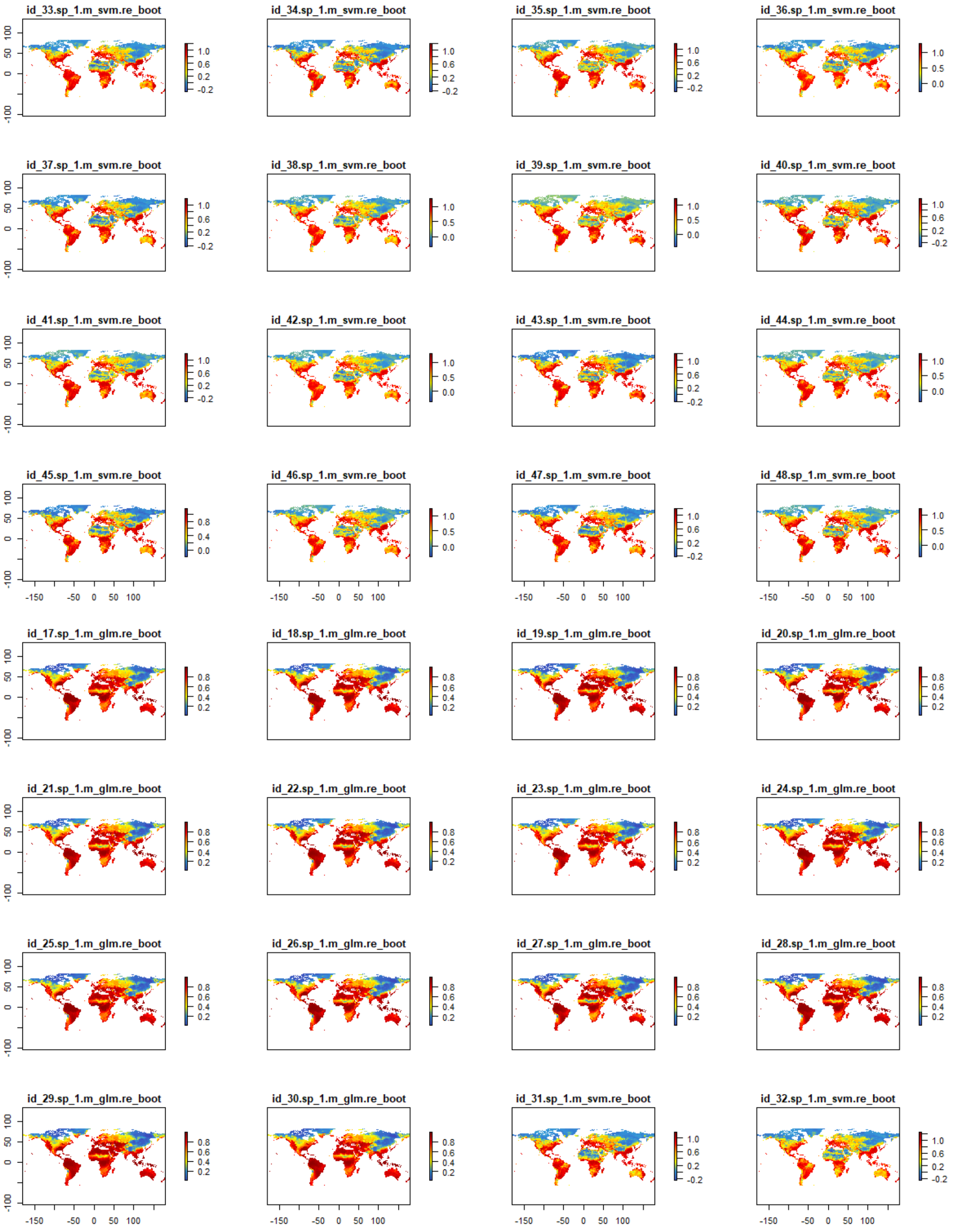

id_33.sp_1.m_svm.re_boot
id_34.sp_1.m_svm.re_boot
id_35.sp_1.m_svm.re_boot
id_36.sp_1.m_svm.re_boot
id_37.sp_1.m_svm.re_boot
id_38.sp_1.m_svm.re_boot
id_39.sp_1.m_svm.re_boot
id_40.sp_1.m_svm.re_boot
id_41.sp_1.m_svm.re_boot
id_42.sp_1.m_svm.re_boot
id_43.sp_1.m_svm.re_boot
id_44.sp_1.m_svm.re_boot
id_45.sp_1.m_svm.re_boot
id_46.sp_1.m_svm.re_boot
id_47.sp_1.m_svm.re_boot
id_48.sp_1.m_svm.re_boot
id_17.sp_1.m_glm.re_boot
id_18.sp_1.m_glm.re_boot
id_19.sp_1.m_glm.re_boot
id_20.sp_1.m_glm.re_boot
id_21.sp_1.m_glm.re_boot
id_22.sp_1.m_glm.re_boot
id_23.sp_1.m_glm.re_boot
id_24.sp_1.m_glm.re_boot
id_25.sp_1.m_glm.re_boot
id_26.sp_1.m_glm.re_boot
id_27.sp_1.m_glm.re_boot
id_28.sp_1.m_glm.re_boot
id_29.sp_1.m_glm.re_boot
id_30.sp_1.m_glm.re_boot
id_31.sp_1.m_svm.re_boot
id_32.sp_1.m_svm.re_boot

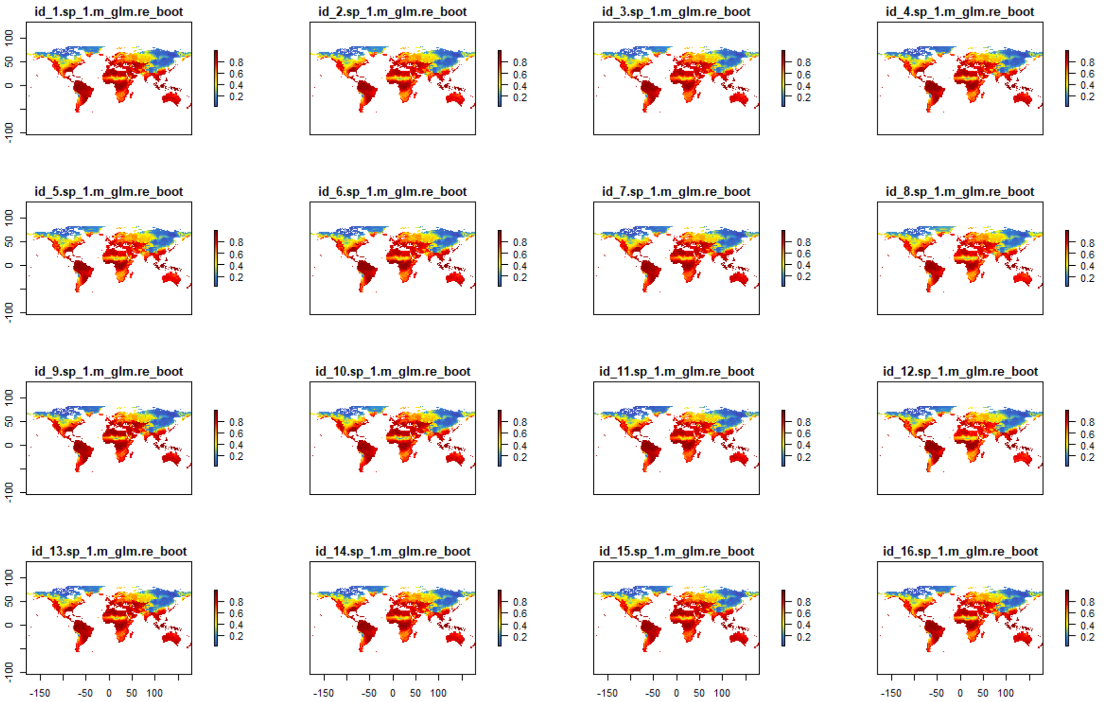
id_1.sp_1.m_glm.re_boot
id_2.sp_1.m_glm.re_boot
id_3.sp_1.m_glm.re_boot
id_4.sp_1.m_glm.re_boot
id_5.sp_1.m_glm.re_boot
id_6.sp_1.m_glm.re_boot
id_7.sp_1.m_glm.re_boot
id_8.sp_1.m_glm.re_boot
id_9.sp_1.m_glm.re_boot
id_10.sp_1.m_glm.re_boot
id_11.sp_1.m_glm.re_boot
id_12.sp_1.m_glm.re_boot
id_13.sp_1.m_glm.re_boot
id_14.sp_1.m_glm.re_boot
id_15.sp_1.m_glm.re_boot
id_16.sp_1.m_glm.re_boot

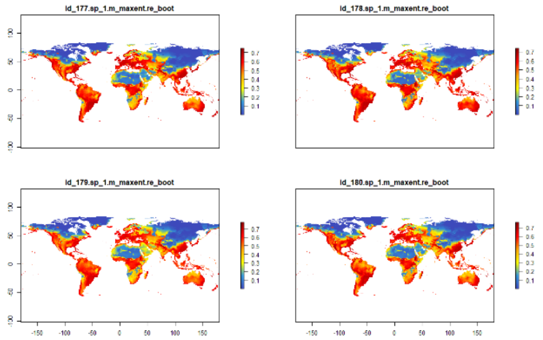
id_177.sp_1.m_maxent.re_boot
id_178.sp_1.m_maxent.re_boot
id_179.sp_1.m_maxent.re_boot
id_180.sp_1.m_maxent.re_boot

Supplementary material I.3. Thirty predictions of potential distribution for RCP 8.5 year 2050 for each algorithm

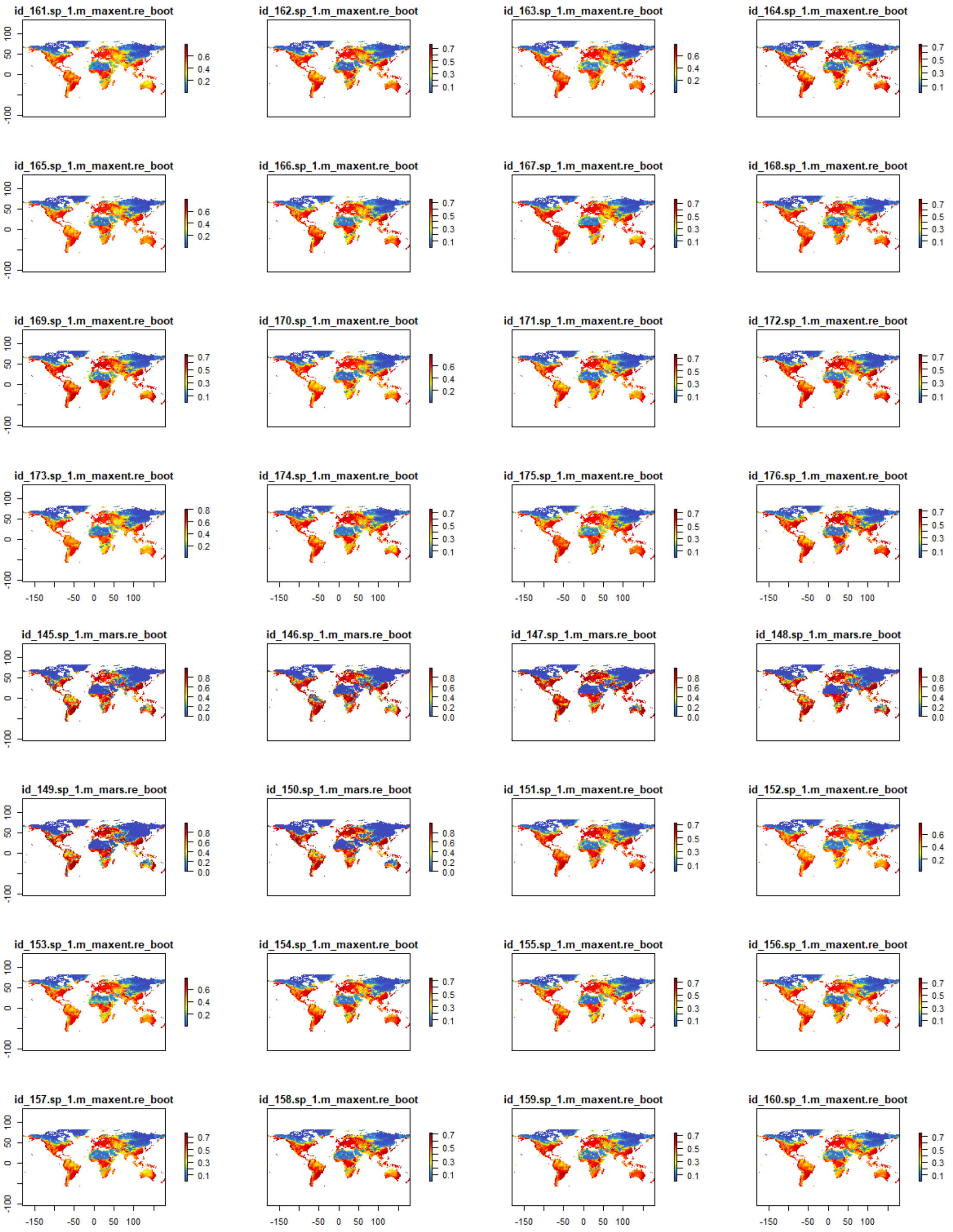

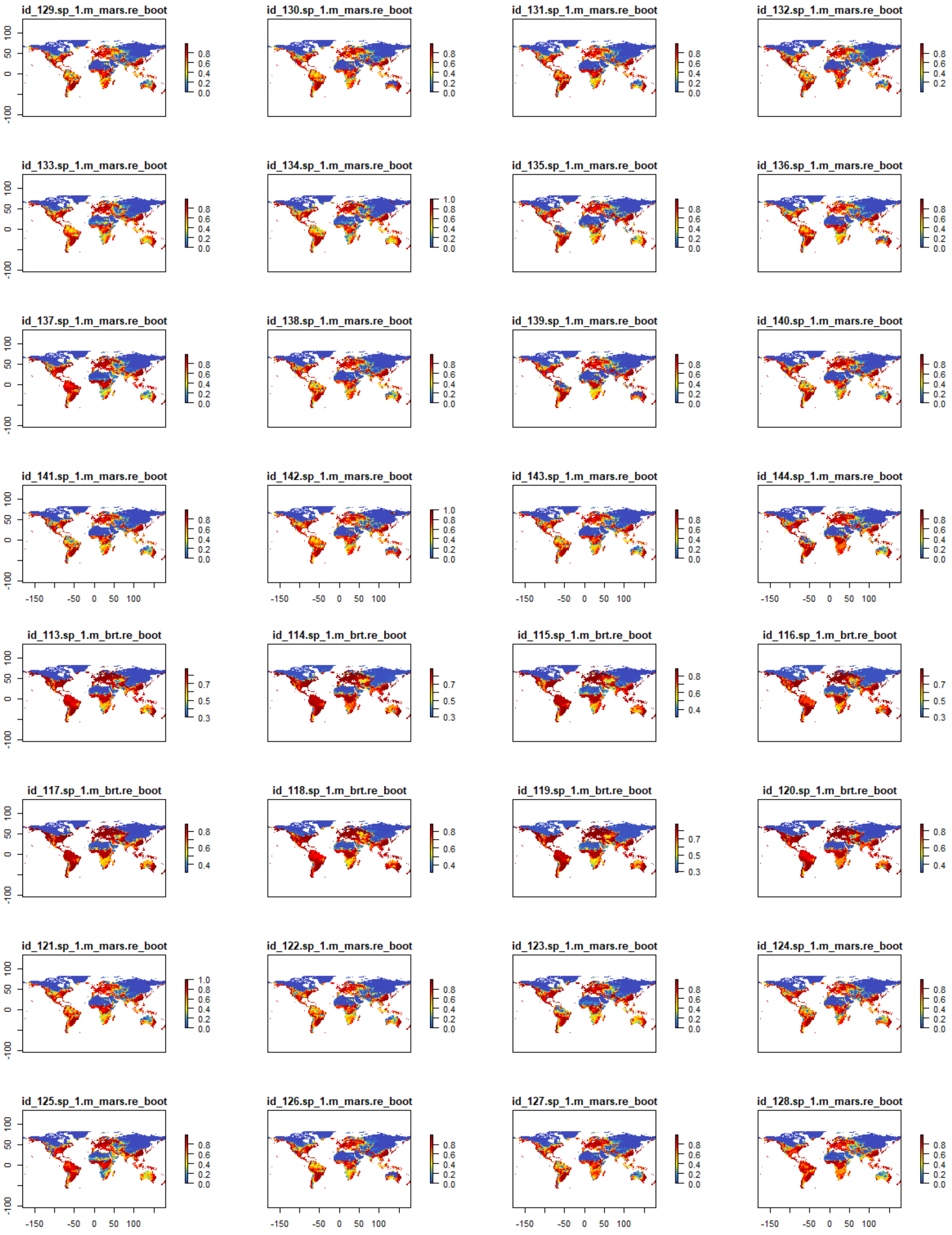
id_129.sp_1.m_mars.re_boot
id_130.sp_1.m_mars.re_boot
id_131.sp_1.m_mars.re_boot
id_132.sp_1.m_mars.re_boot
id_133.sp_1.m_mars.re_boot
id_134.sp_1.m_mars.re_boot
id_135.sp_1.m_mars.re_boot
id_136.sp_1.m_mars.re_boot
id_137.sp_1.m_mars.re_boot
id_138.sp_1.m_mars.re_boot
id_139.sp_1.m_mars.re_boot
id_140.sp_1.m_mars.re_boot
id_141.sp_1.m_mars.re_boot
id_142.sp_1.m_mars.re_boot
id_143.sp_1.m_mars.re_boot
id_144.sp_1.m_mars.re_boot
id_113.sp_1.m_brt.re_boot
id_114.sp_1.m_brt.re_boot
id_115.sp_1.m_brt.re_boot
id_116.sp_1.m_brt.re_boot
id_117.sp_1.m_brt.re_boot
id_118.sp_1.m_brt.re_boot
id_119.sp_1.m_brt.re_boot
id_120.sp_1.m_brt.re_boot
id_121.sp_1.m_mars.re_boot
id_122.sp_1.m_mars.re_boot
id_123.sp_1.m_mars.re_boot
id_124.sp_1.m_mars.re_boot
id_125.sp_1.m_mars.re_boot
id_126.sp_1.m_mars.re_boot
id_127.sp_1.m_mars.re_boot
id_128.sp_1.m_mars.re_boot

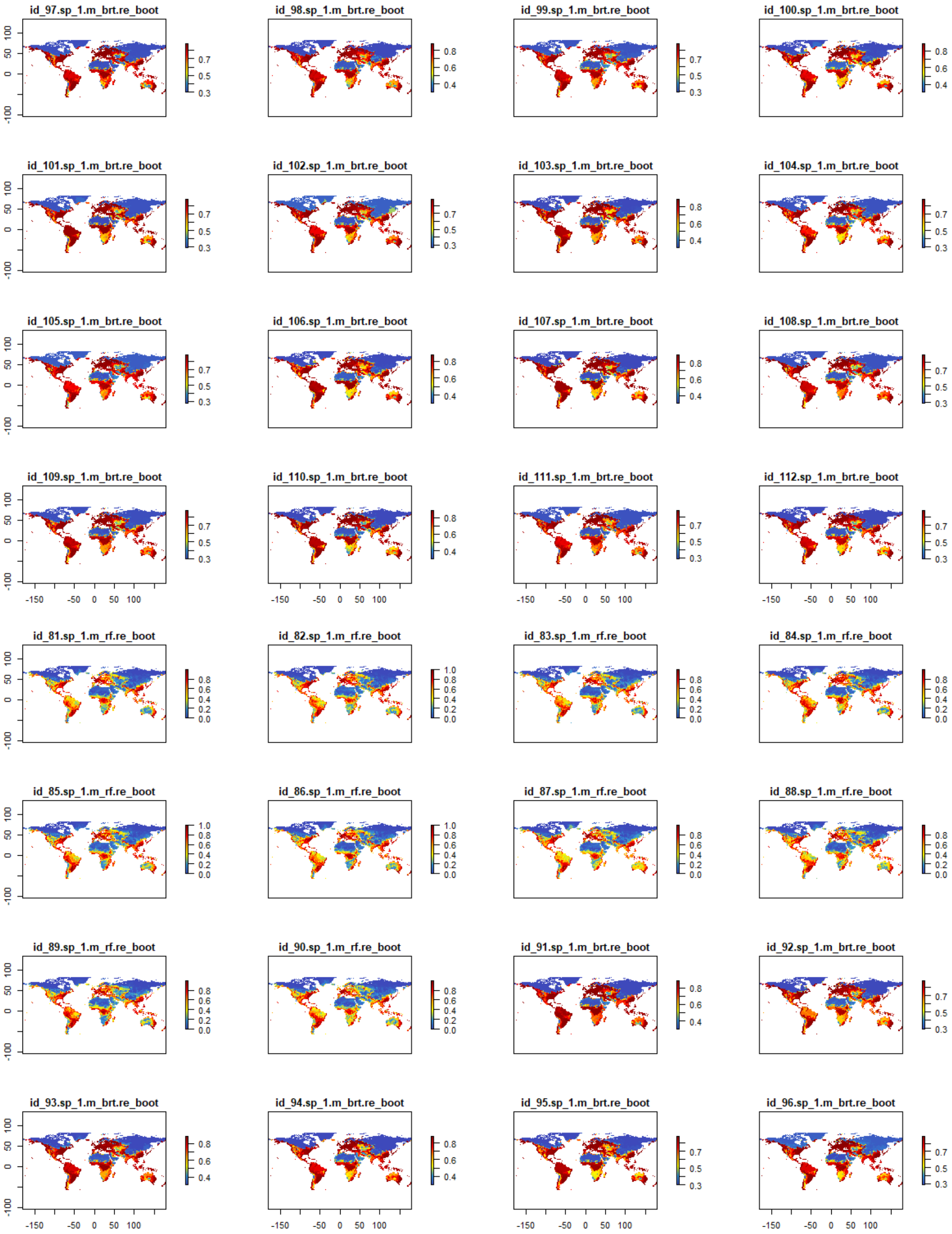
id_97.sp_1.m_brt.re_boot
id_98.sp_1.m_brt.re_boot
id_99.sp_1.m_brt.re_boot
id_100.sp_1.m_brt.re_boot
id_101.sp_1.m_brt.re_boot
id_102.sp_1.m_brt.re_boot
id_103.sp_1.m_brt.re_boot
id_104.sp_1.m_brt.re_boot
id_105.sp_1.m_brt.re_boot
id_106.sp_1.m_brt.re_boot
id_107.sp_1.m_brt.re_boot
id_108.sp_1.m_brt.re_boot
id_109.sp_1.m_brt.re_boot
id_110.sp_1.m_brt.re_boot
id_111.sp_1.m_brt.re_boot
id_112.sp_1.m_brt.re_boot
id_81.sp_1.m_rf.re_boot
id_82.sp_1.m_rf.re_boot
id_83.sp_1.m_rf.re_boot
id_84.sp_1.m_rf.re_boot
id_85.sp_1.m_rf.re_boot
id_86.sp_1.m_rf.re_boot
id_87.sp_1.m_rf.re_boot
id_88.sp_1.m_rf.re_boot
id_89.sp_1.m_rf.re_boot
id_90.sp_1.m_rf.re_boot
id_91.sp_1.m_brt.re_boot
id_92.sp_1.m_brt.re_boot
id_93.sp_1.m_brt.re_boot
id_94.sp_1.m_brt.re_boot
id_95.sp_1.m_brt.re_boot
id_96.sp_1.m_brt.re_boot

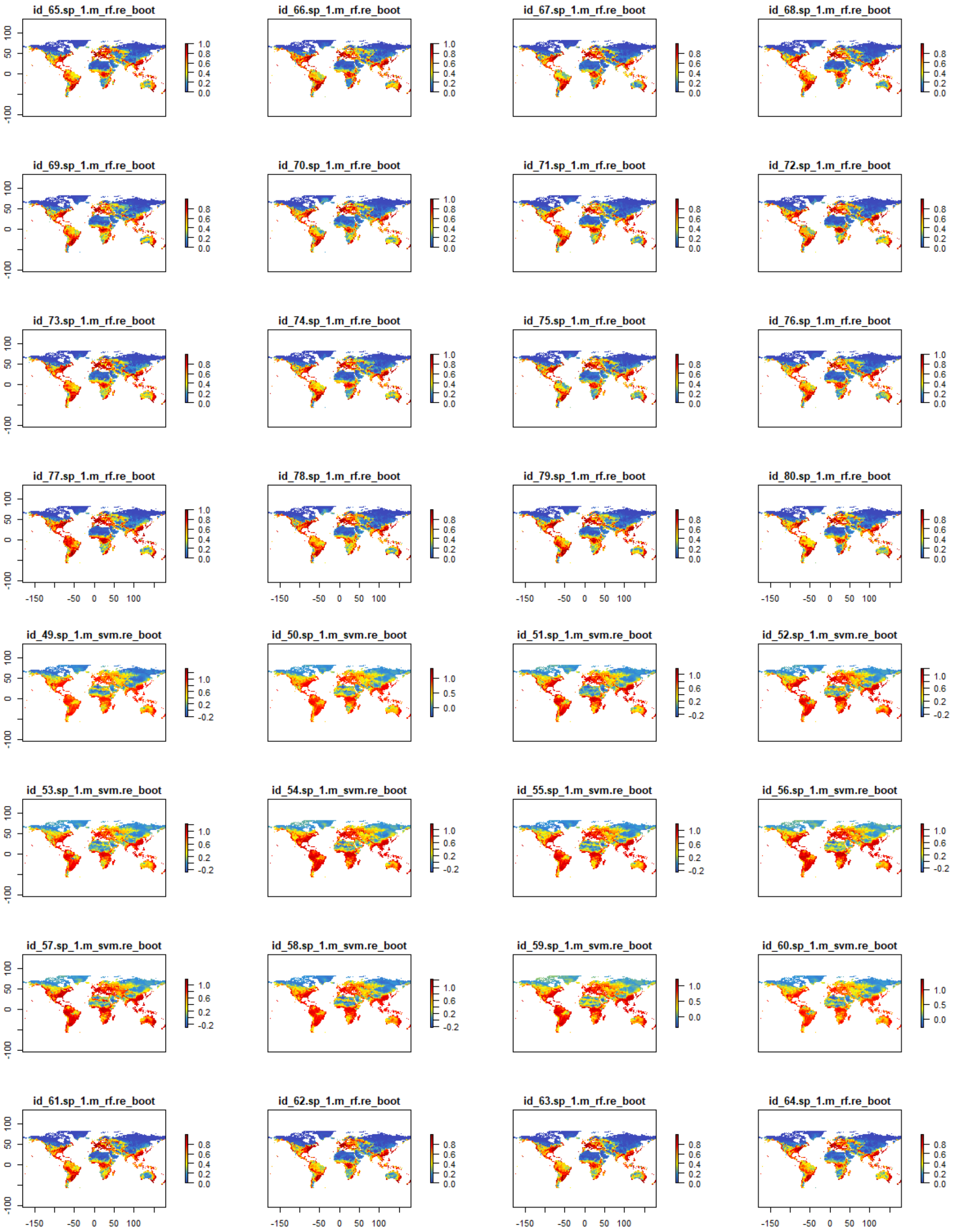
id_65.sp_1.m_rf.re_boot
id_66.sp_1.m_rf.re_boot
id_67.sp_1.m_rf.re_boot
id_68.sp_1.m_rf.re_boot
id_69.sp_1.m_rf.re_boot
id_70.sp_1.m_rf.re_boot
id_71.sp_1.m_rf.re_boot
id_72.sp_1.m_rf.re_boot
id_73.sp_1.m_rf.re_boot
id_74.sp_1.m_rf.re_boot
id_75.sp_1.m_rf.re_boot
id_76.sp_1.m_rf.re_boot
id_77.sp_1.m_rf.re_boot
id_78.sp_1.m_rf.re_boot
id_79.sp_1.m_rf.re_boot
id_80.sp_1.m_rf.re_boot
id_49.sp_1.m_svm.re_boot
id_50.sp_1.m_svm.re_boot
id_51.sp_1.m_svm.re_boot
id_52.sp_1.m_svm.re_boot
id_53.sp_1.m_svm.re_boot
id_54.sp_1.m_svm.re_boot
id_55.sp_1.m_svm.re_boot
id_56.sp_1.m_svm.re_boot
id_57.sp_1.m_svm.re_boot
id_58.sp_1.m_svm.re_boot
id_59.sp_1.m_svm.re_boot
id_60.sp_1.m_svm.re_boot
id_61.sp_1.m_rf.re_boot
id_62.sp_1.m_rf.re_boot
id_63.sp_1.m_rf.re_boot
id_64.sp_1.m_rf.re_boot

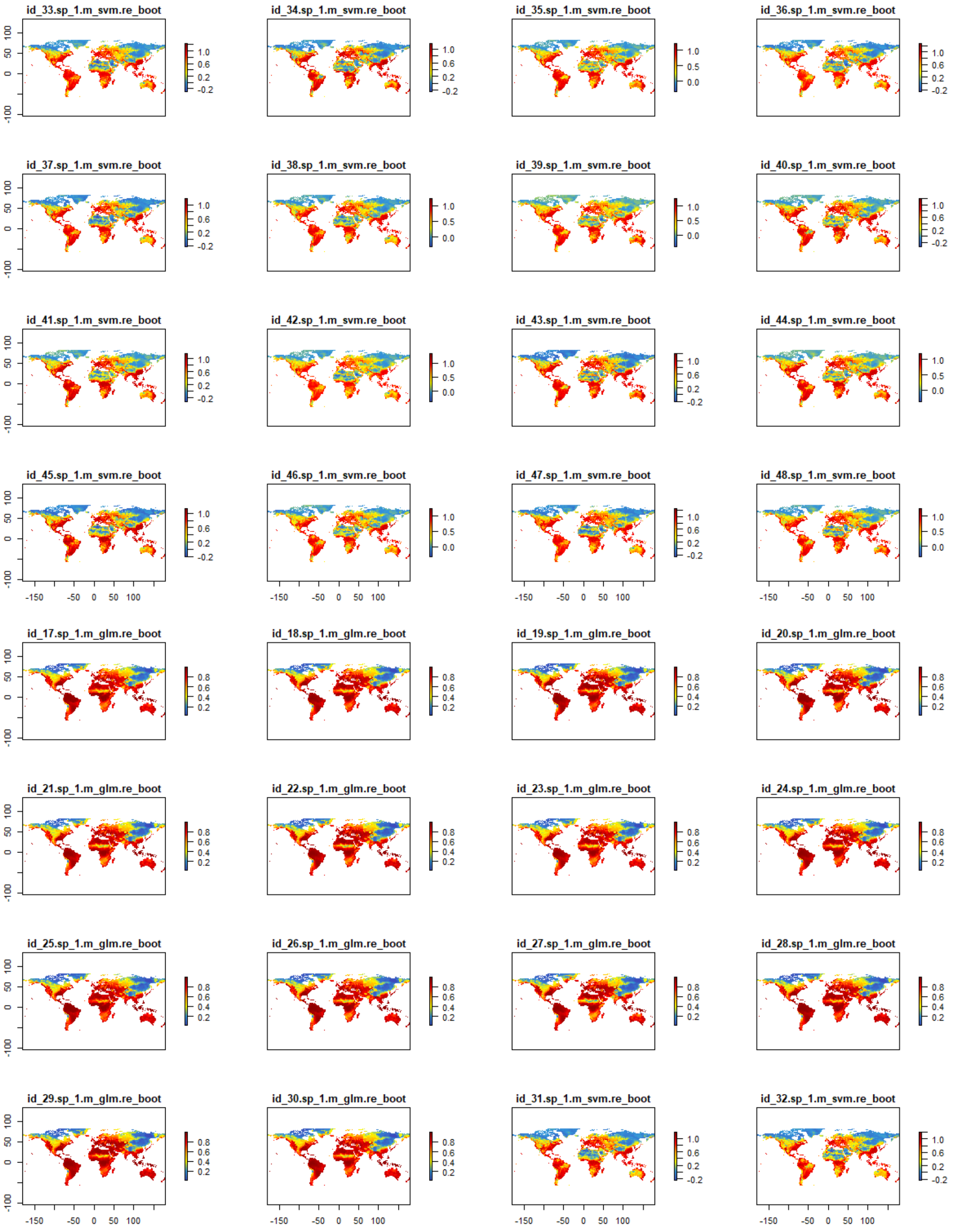
id_33.sp_1.m_svm.re_boot
id_34.sp_1.m_svm.re_boot
id_35.sp_1.m_svm.re_boot
id_36.sp_1.m_svm.re_boot
id_37.sp_1.m_svm.re_boot
id_38.sp_1.m_svm.re_boot
id_39.sp_1.m_svm.re_boot
id_40.sp_1.m_svm.re_boot
id_41.sp_1.m_svm.re_boot
id_42.sp_1.m_svm.re_boot
id_43.sp_1.m_svm.re_boot
id_44.sp_1.m_svm.re_boot
id_45.sp_1.m_svm.re_boot
id_46.sp_1.m_svm.re_boot
id_47.sp_1.m_svm.re_boot
id_48.sp_1.m_svm.re_boot
id_17.sp_1.m_glm.re_boot
id_18.sp_1.m_glm.re_boot
id_19.sp_1.m_glm.re_boot
id_20.sp_1.m_glm.re_boot
id_21.sp_1.m_glm.re_boot
id_22.sp_1.m_glm.re_boot
id_23.sp_1.m_glm.re_boot
id_24.sp_1.m_glm.re_boot
id_25.sp_1.m_glm.re_boot
id_26.sp_1.m_glm.re_boot
id_27.sp_1.m_glm.re_boot
id_28.sp_1.m_glm.re_boot
id_29.sp_1.m_glm.re_boot
id_30.sp_1.m_glm.re_boot
id_31.sp_1.m_svm.re_boot
id_32.sp_1.m_svm.re_boot

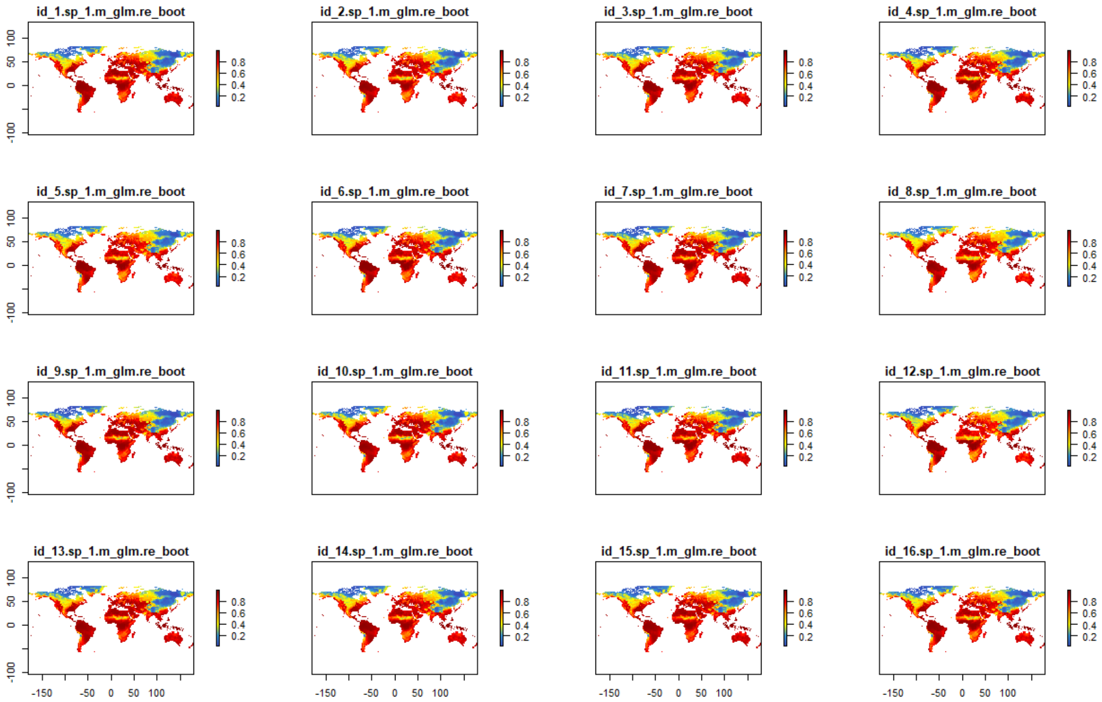
id_1.sp_1.m_glm.re_boot
id_2.sp_1.m_glm.re_boot
id_3.sp_1.m_glm.re_boot
id_4.sp_1.m_glm.re_boot
id_5.sp_1.m_glm.re_boot
id_6.sp_1.m_glm.re_boot
id_7.sp_1.m_glm.re_boot
id_8.sp_1.m_glm.re_boot
id_9.sp_1.m_glm.re_boot
id_10.sp_1.m_glm.re_boot
id_11.sp_1.m_glm.re_boot
id_12.sp_1.m_glm.re_boot
id_13.sp_1.m_glm.re_boot
id_14.sp_1.m_glm.re_boot
id_15.sp_1.m_glm.re_boot
id_16.sp_1.m_glm.re_boot

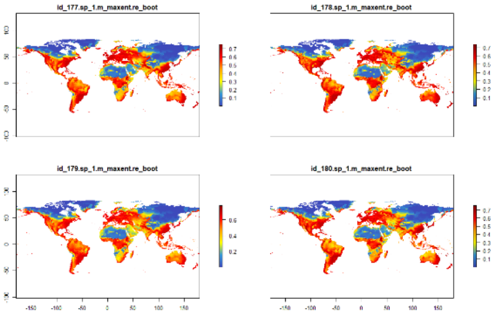
id_177.sp_1.m_maxent.re_boot
id_178.sp_1.m_maxent.re_boot
id_179.sp_1.m_maxent.re_boot
id_180.sp_1.m_maxent.re_boot

Supplementary material I.4. Thirty predictions of potential distribution for RCP 4.5 year 2070 for each algorithm

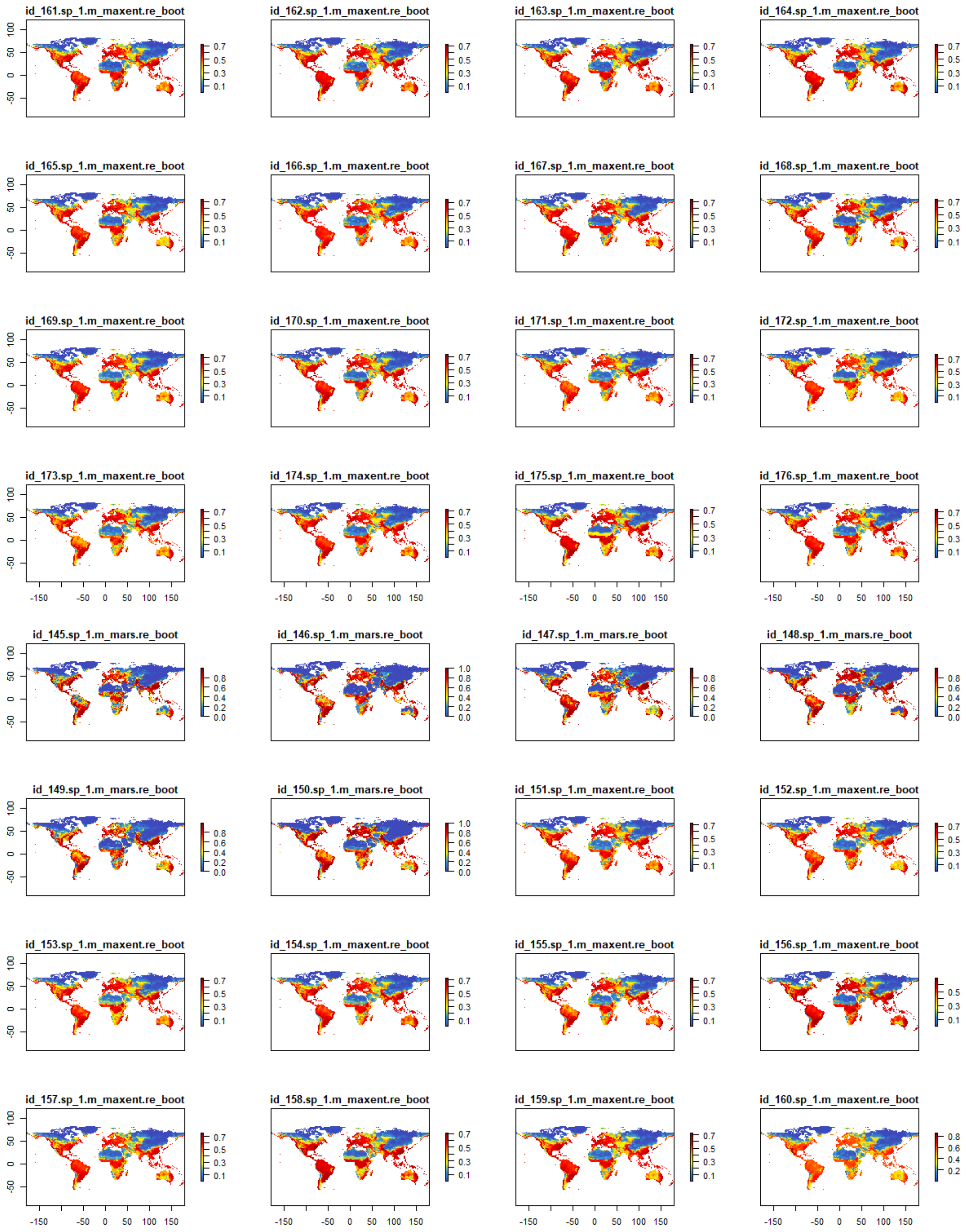

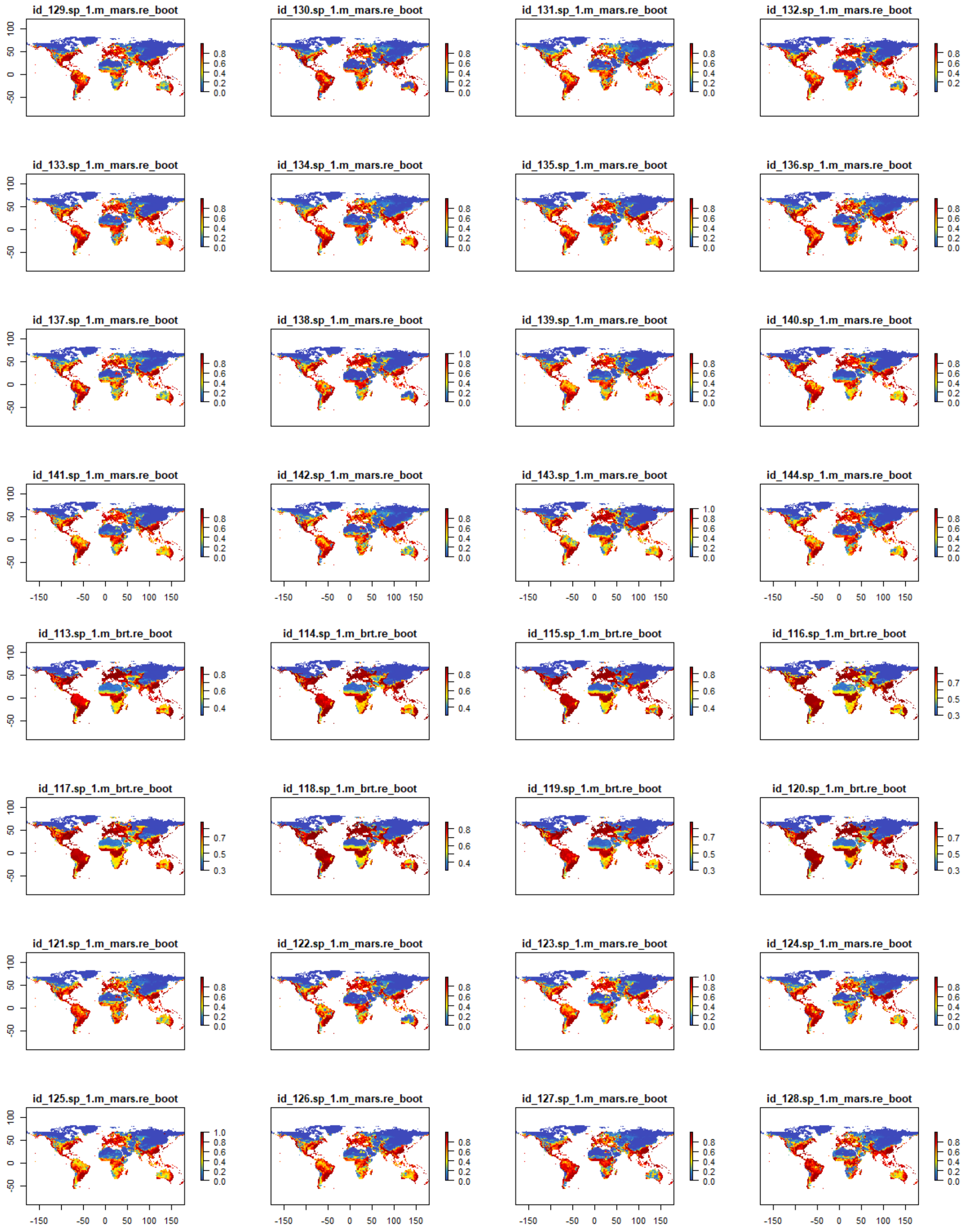
id_129.sp_1.m_mars.re_boot
id_130.sp_1.m_mars.re_boot
id_131.sp_1.m_mars.re_boot
id_132.sp_1.m_mars.re_boot
id_133.sp_1.m_mars.re_boot
id_134.sp_1.m_mars.re_boot
id_135.sp_1.m_mars.re_boot
id_136.sp_1.m_mars.re_boot
id_137.sp_1.m_mars.re_boot
id_138.sp_1.m_mars.re_boot
id_139.sp_1.m_mars.re_boot
id_140.sp_1.m_mars.re_boot
id_141.sp_1.m_mars.re_boot
id_142.sp_1.m_mars.re_boot
id_143.sp_1.m_mars.re_boot
id_144.sp_1.m_mars.re_boot
id_113.sp_1.m_brt.re_boot
id_114.sp_1.m_brt.re_boot
id_115.sp_1.m_brt.re_boot
id_116.sp_1.m_brt.re_boot
id_117.sp_1.m_brt.re_boot
id_118.sp_1.m_brt.re_boot
id_119.sp_1.m_brt.re_boot
id_120.sp_1.m_brt.re_boot
id_121.sp_1.m_mars.re_boot
id_122.sp_1.m_mars.re_boot
id_123.sp_1.m_mars.re_boot
id_124.sp_1.m_mars.re_boot
id_125.sp_1.m_mars.re_boot
id_126.sp_1.m_mars.re_boot
id_127.sp_1.m_mars.re_boot
id_128.sp_1.m_mars.re_boot

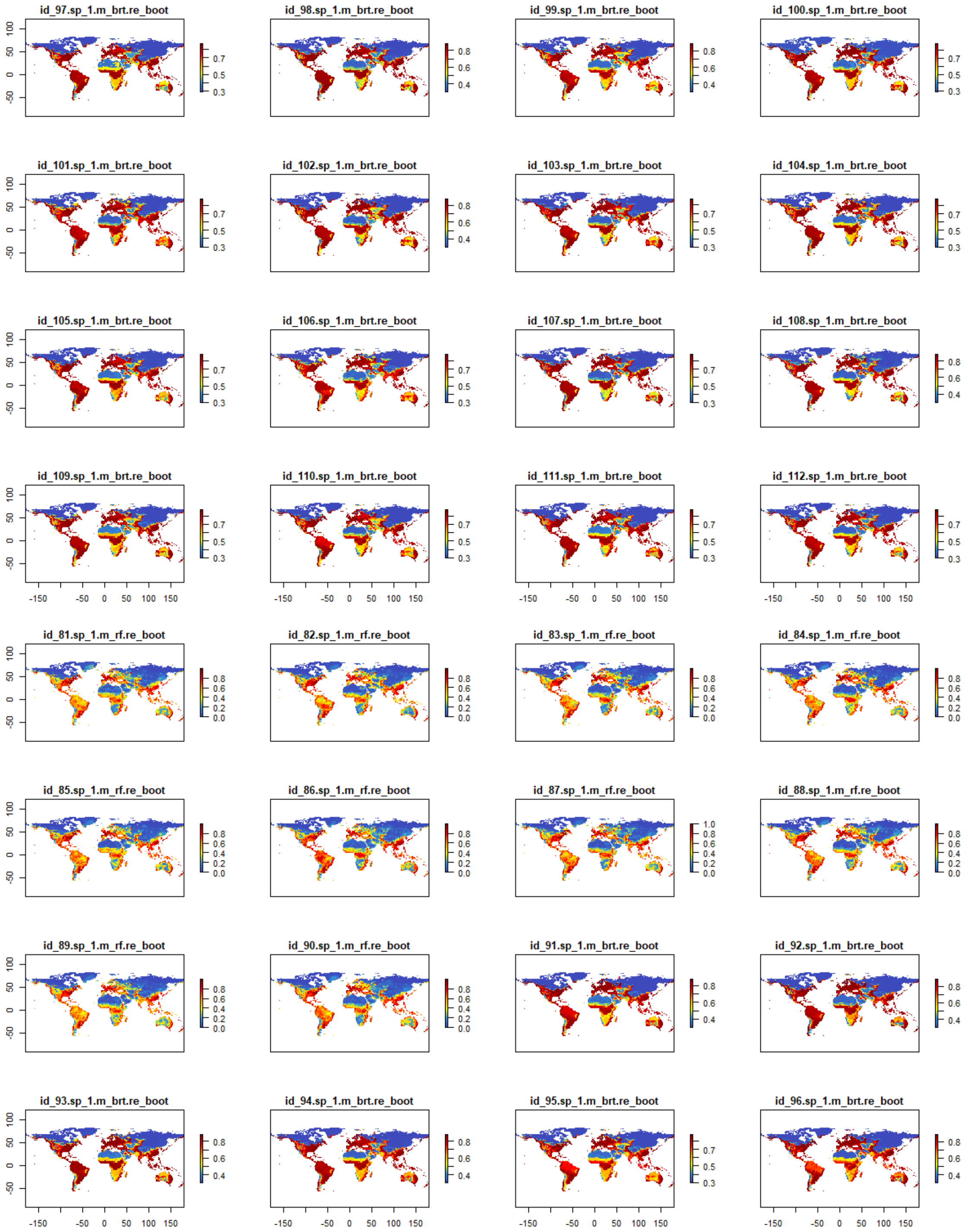
id_97.sp_1.m_brt.re_boot
id_98.sp_1.m_brt.re_boot
id_99.sp_1.m_brt.re_boot
id_100.sp_1.m_brt.re_boot
id_101.sp_1.m_brt.re_boot
id_102.sp_1.m_brt.re_boot
id_103.sp_1.m_brt.re_boot
id_104.sp_1.m_brt.re_boot
id_105.sp_1.m_brt.re_boot
id_106.sp_1.m_brt.re_boot
id_107.sp_1.m_brt.re_boot
id_108.sp_1.m_brt.re_boot
id_109.sp_1.m_brt.re_boot
id_110.sp_1.m_brt.re_boot
id_111.sp_1.m_brt.re_boot
id_112.sp_1.m_brt.re_boot
id_81.sp_1.m_rf.re_boot
id_82.sp_1.m_rf.re_boot
id_83.sp_1.m_rf.re_boot
id_84.sp_1.m_rf.re_boot
id_85.sp_1.m_rf.re_boot
id_86.sp_1.m_rf.re_boot
id_87.sp_1.m_rf.re_boot
id_88.sp_1.m_rf.re_boot
id_89.sp_1.m_rf.re_boot
id_90.sp_1.m_rf.re_boot
id_91.sp_1.m_brt.re_boot
id_92.sp_1.m_brt.re_boot
id_93.sp_1.m_brt.re_boot
id_94.sp_1.m_brt.re_boot
id_95.sp_1.m_brt.re_boot
id_96.sp_1.m_brt.re_boot

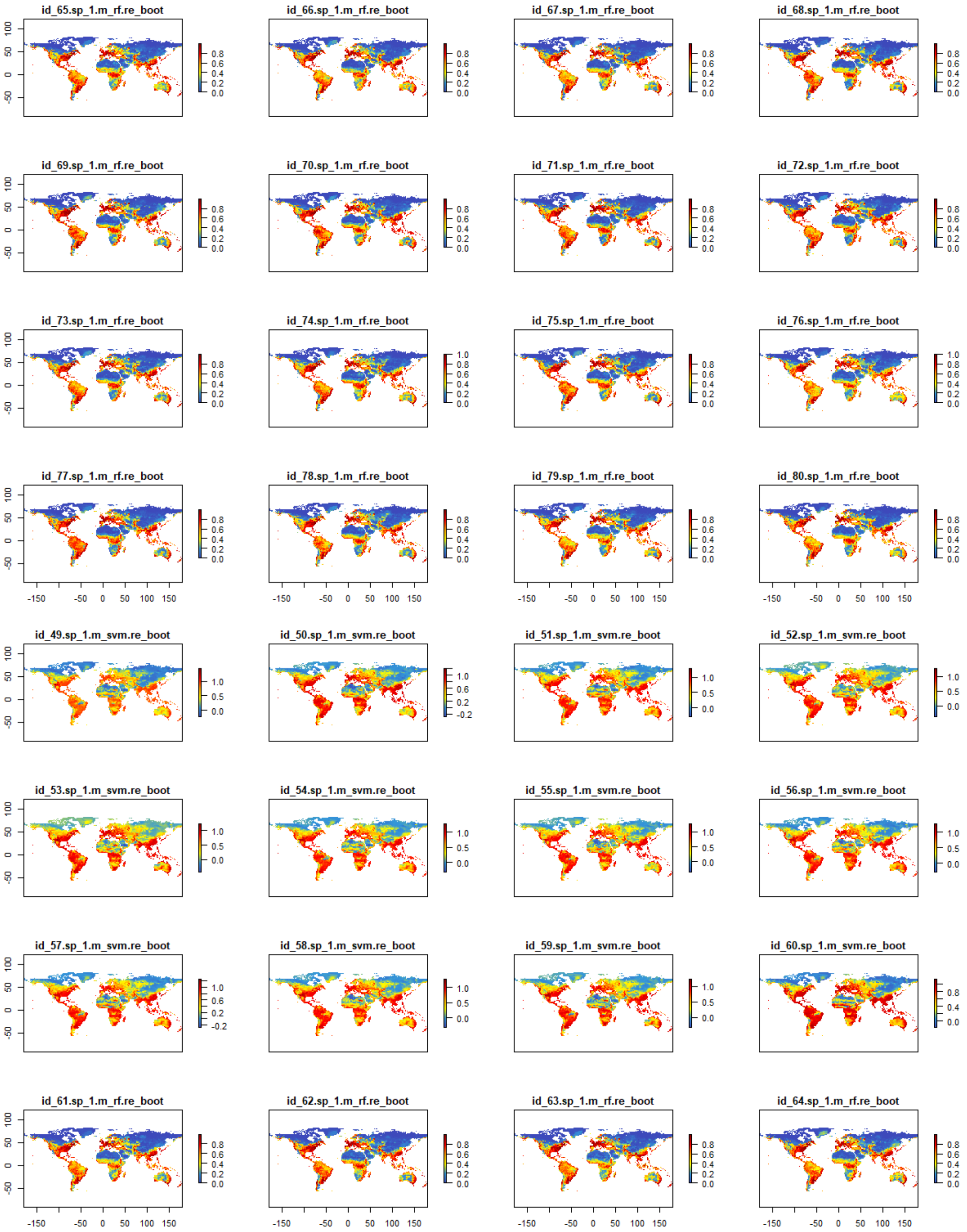
id_65.sp_1.m_rf.re_boot
id_66.sp_1.m_rf.re_boot
id_67.sp_1.m_rf.re_boot
id_68.sp_1.m_rf.re_boot
id_69.sp_1.m_rf.re_boot
id_70.sp_1.m_rf.re_boot
id_71.sp_1.m_rf.re_boot
id_72.sp_1.m_rf.re_boot
id_73.sp_1.m_rf.re_boot
id_74.sp_1.m_rf.re_boot
id_75.sp_1.m_rf.re_boot
id_76.sp_1.m_rf.re_boot
id_77.sp_1.m_rf.re_boot
id_78.sp_1.m_rf.re_boot
id_79.sp_1.m_rf.re_boot
id_80.sp_1.m_rf.re_boot
id_49.sp_1.m_svm.re_boot
id_50.sp_1.m_svm.re_boot
id_51.sp_1.m_svm.re_boot
id_52.sp_1.m_svm.re_boot
id_53.sp_1.m_svm.re_boot
id_54.sp_1.m_svm.re_boot
id_55.sp_1.m_svm.re_boot
id_56.sp_1.m_svm.re_boot
id_57.sp_1.m_svm.re_boot
id_58.sp_1.m_svm.re_boot
id_59.sp_1.m_svm.re_boot
id_60.sp_1.m_svm.re_boot
id_61.sp_1.m_rf.re_boot
id_62.sp_1.m_rf.re_boot
id_63.sp_1.m_rf.re_boot
id_64.sp_1.m_rf.re_boot

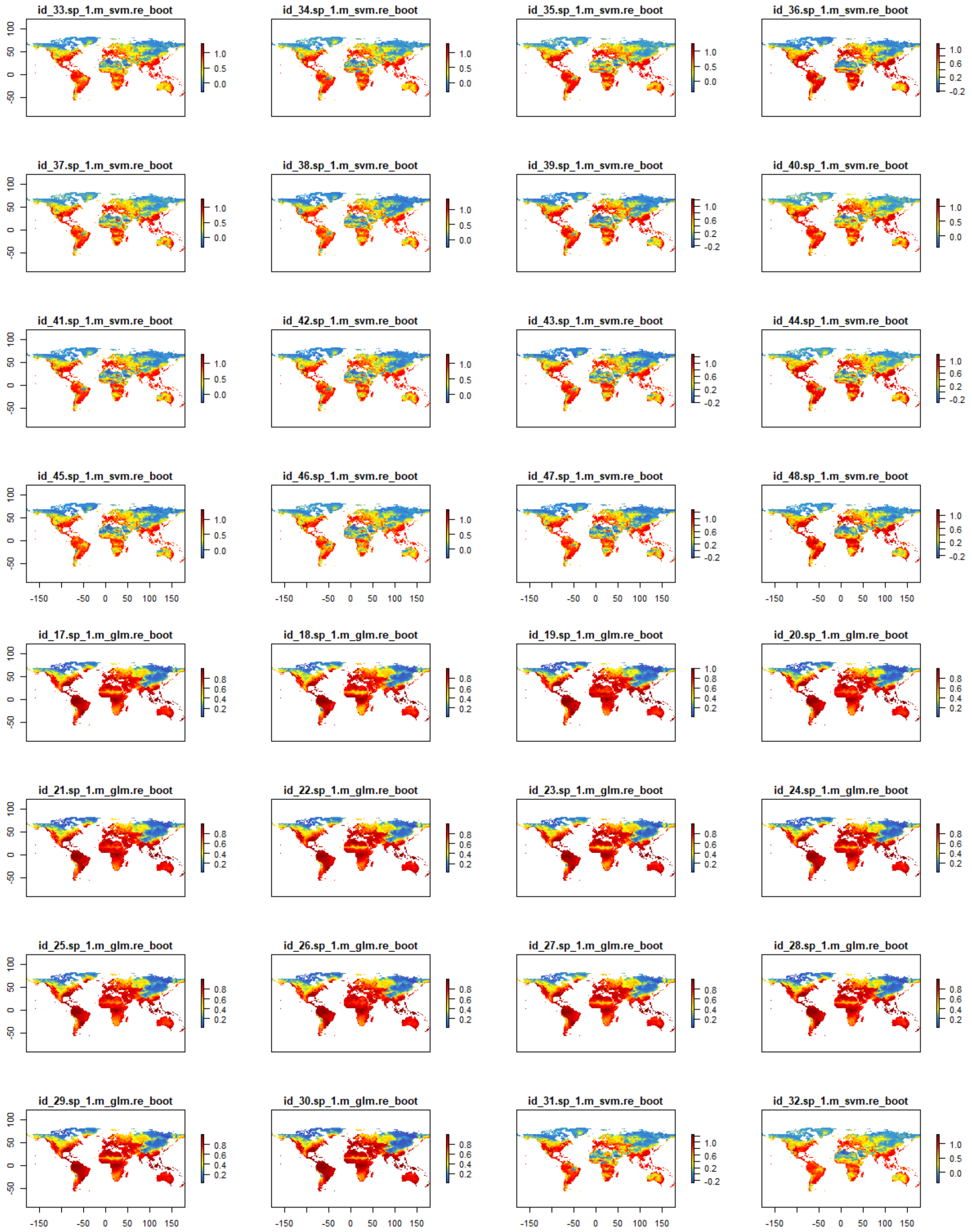
id_33.sp_1.m_svm.re_boot
id_34.sp_1.m_svm.re_boot
id_35.sp_1.m_svm.re_boot
id_36.sp_1.m_svm.re_boot
id_37.sp_1.m_svm.re_boot
id_38.sp_1.m_svm.re_boot
id_39.sp_1.m_svm.re_boot
id_40.sp_1.m_svm.re_boot
id_41.sp_1.m_svm.re_boot
id_42.sp_1.m_svm.re_boot
id_43.sp_1.m_svm.re_boot
id_44.sp_1.m_svm.re_boot
id_45.sp_1.m_svm.re_boot
id_46.sp_1.m_svm.re_boot
id_47.sp_1.m_svm.re_boot
id_48.sp_1.m_svm.re_boot
id_17.sp_1.m_glm.re_boot
id_18.sp_1.m_glm.re_boot
id_19.sp_1.m_glm.re_boot
id_20.sp_1.m_glm.re_boot
id_21.sp_1.m_glm.re_boot
id_22.sp_1.m_glm.re_boot
id_23.sp_1.m_glm.re_boot
id_24.sp_1.m_glm.re_boot
id_25.sp_1.m_glm.re_boot
id_26.sp_1.m_glm.re_boot
id_27.sp_1.m_glm.re_boot
id_28.sp_1.m_glm.re_boot
id_29.sp_1.m_glm.re_boot
id_30.sp_1.m_glm.re_boot
id_31.sp_1.m_svm.re_boot
id_32.sp_1.m_svm.re_boot
-150 -50 0 50 100 150
-50 0 50 100

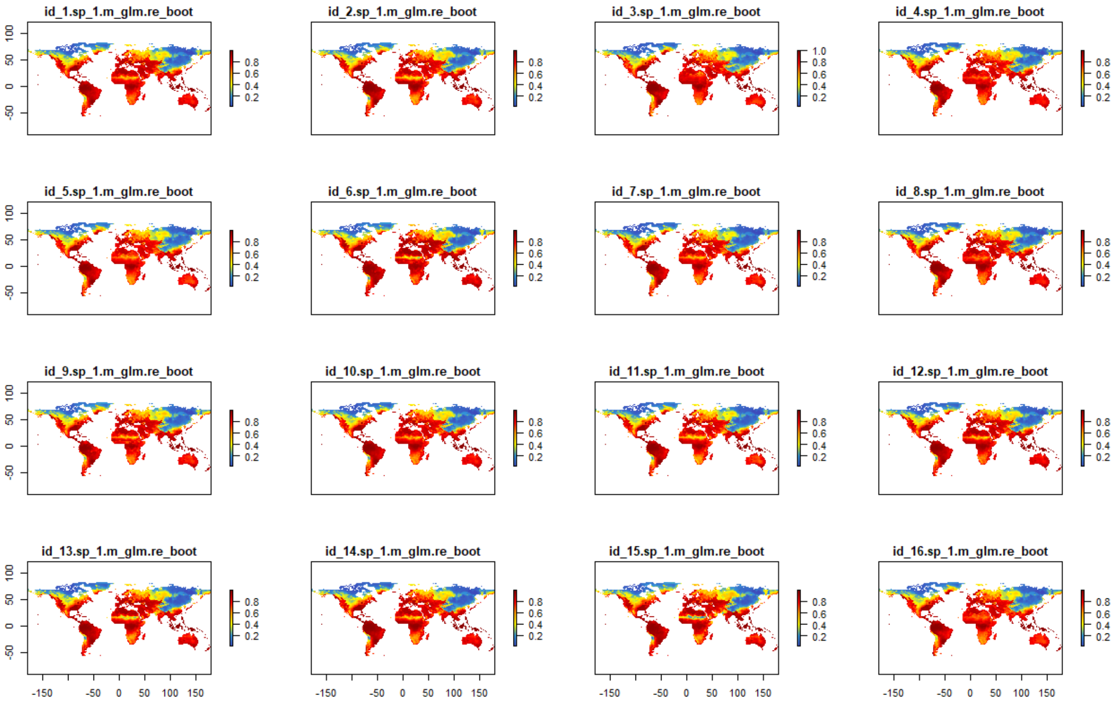
id_1.sp_1.m_glm.re_boot
id_2.sp_1.m_glm.re_boot
id_3.sp_1.m_glm.re_boot
id_4.sp_1.m_glm.re_boot
id_5.sp_1.m_glm.re_boot
id_6.sp_1.m_glm.re_boot
id_7.sp_1.m_glm.re_boot
id_8.sp_1.m_glm.re_boot
id_9.sp_1.m_glm.re_boot
id_10.sp_1.m_glm.re_boot
id_11.sp_1.m_glm.re_boot
id_12.sp_1.m_glm.re_boot
id_13.sp_1.m_glm.re_boot
id_14.sp_1.m_glm.re_boot
id_15.sp_1.m_glm.re_boot
id_16.sp_1.m_glm.re_boot

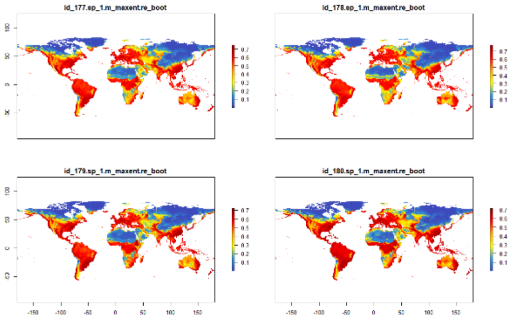
id_177.sp_1.m_maxent.re_boot
id_178.sp_1.m_maxent.re_boot
id_179.sp_1.m_maxent.re_boot
id_180.sp_1.m_maxent.re_boot

Supplementary material I.5. Thirty predictions of potential distribution for RCP 8.5 year 2070 for each algorithm

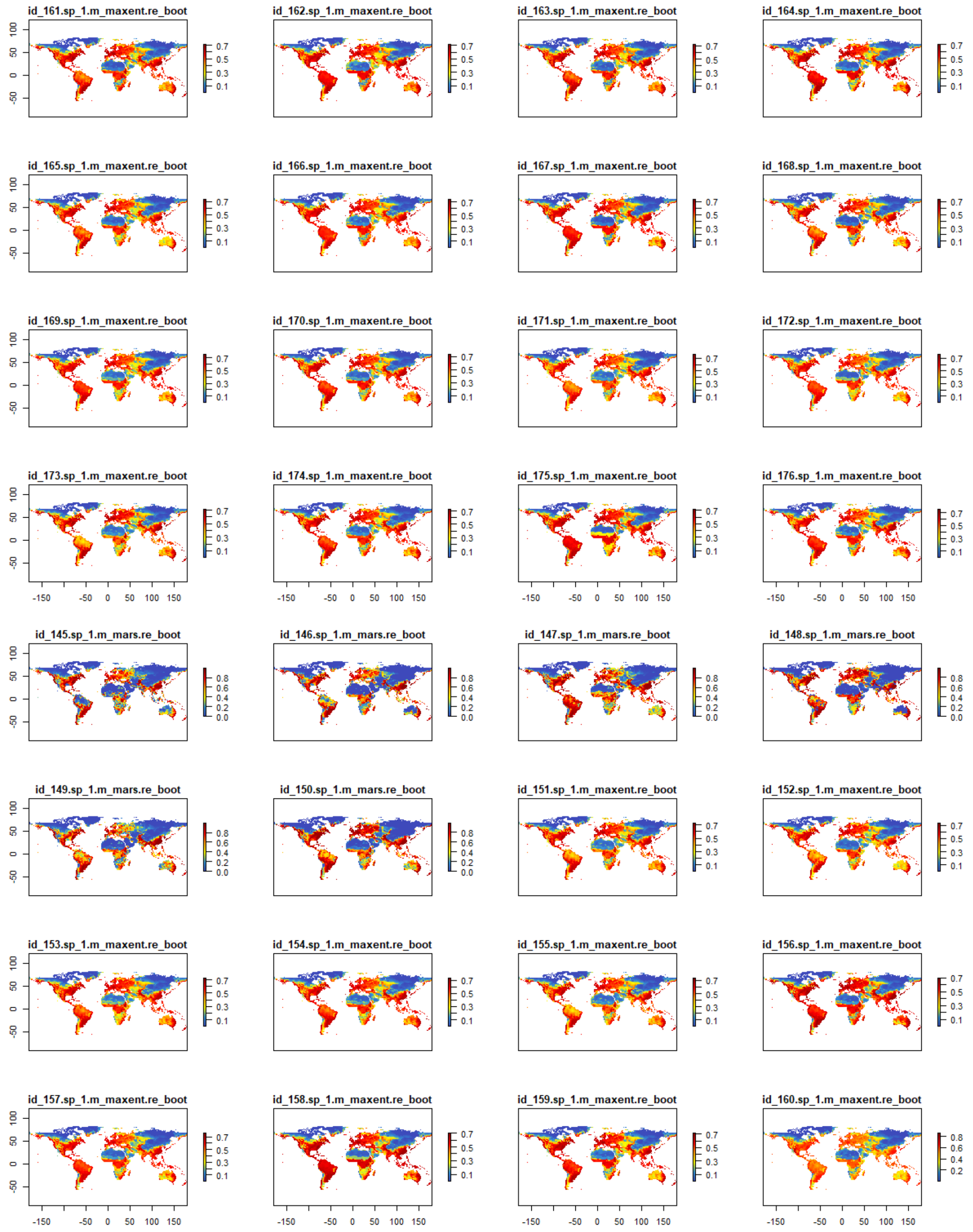

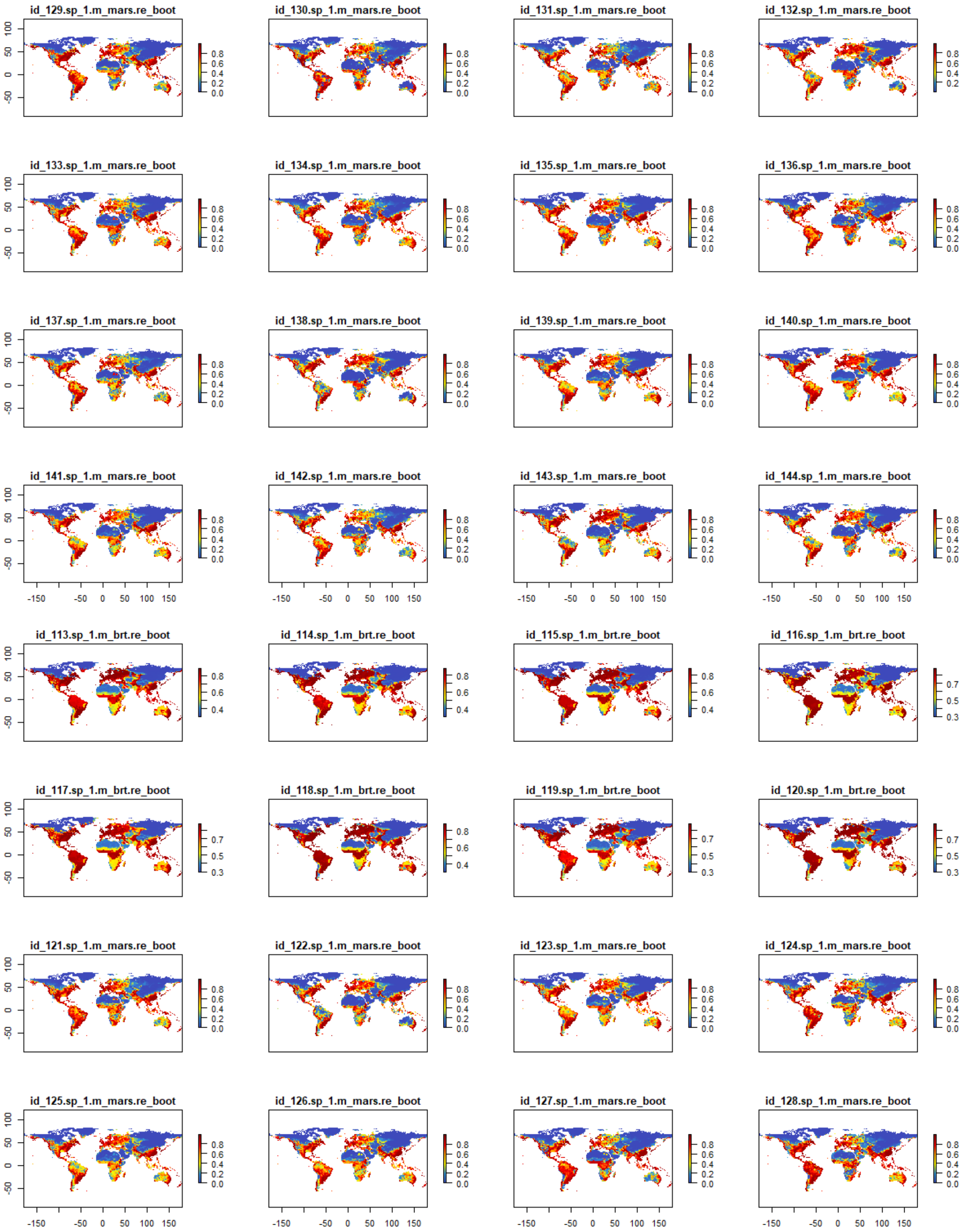
id_129.sp_1.m_mars.re_boot
id_130.sp_1.m_mars.re_boot
id_131.sp_1.m_mars.re_boot
id_132.sp_1.m_mars.re_boot
id_133.sp_1.m_mars.re_boot
id_134.sp_1.m_mars.re_boot
id_135.sp_1.m_mars.re_boot
id_136.sp_1.m_mars.re_boot
id_137.sp_1.m_mars.re_boot
id_138.sp_1.m_mars.re_boot
id_139.sp_1.m_mars.re_boot
id_140.sp_1.m_mars.re_boot
id_141.sp_1.m_mars.re_boot
id_142.sp_1.m_mars.re_boot
id_143.sp_1.m_mars.re_boot
id_144.sp_1.m_mars.re_boot
id_113.sp_1.m_brt.re_boot
id_114.sp_1.m_brt.re_boot
id_115.sp_1.m_brt.re_boot
id_116.sp_1.m_brt.re_boot
id_117.sp_1.m_brt.re_boot
id_118.sp_1.m_brt.re_boot
id_119.sp_1.m_brt.re_boot
id_120.sp_1.m_brt.re_boot
id_121.sp_1.m_mars.re_boot
id_122.sp_1.m_mars.re_boot
id_123.sp_1.m_mars.re_boot
id_124.sp_1.m_mars.re_boot
id_125.sp_1.m_mars.re_boot
id_126.sp_1.m_mars.re_boot
id_127.sp_1.m_mars.re_boot
id_128.sp_1.m_mars.re_boot

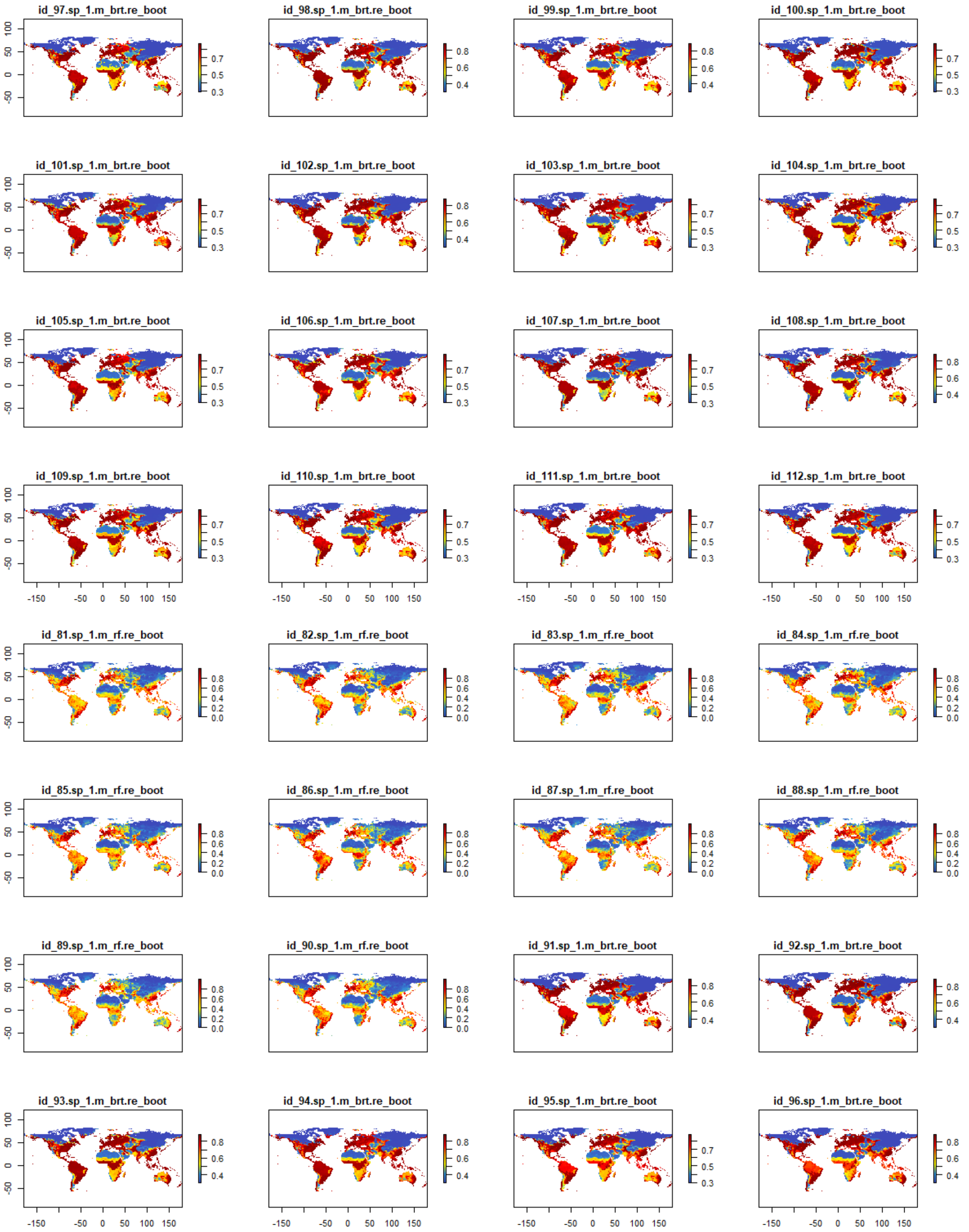
id_97.sp_1.m_brt.re_boot
id_98.sp_1.m_brt.re_boot
id_99.sp_1.m_brt.re_boot
id_100.sp_1.m_brt.re_boot
id_101.sp_1.m_brt.re_boot
id_102.sp_1.m_brt.re_boot
id_103.sp_1.m_brt.re_boot
id_104.sp_1.m_brt.re_boot
id_105.sp_1.m_brt.re_boot
id_106.sp_1.m_brt.re_boot
id_107.sp_1.m_brt.re_boot
id_108.sp_1.m_brt.re_boot
id_109.sp_1.m_brt.re_boot
id_110.sp_1.m_brt.re_boot
id_111.sp_1.m_brt.re_boot
id_112.sp_1.m_brt.re_boot
id_81.sp_1.m_rf.re_boot
id_82.sp_1.m_rf.re_boot
id_83.sp_1.m_rf.re_boot
id_84.sp_1.m_rf.re_boot
id_85.sp_1.m_rf.re_boot
id_86.sp_1.m_rf.re_boot
id_87.sp_1.m_rf.re_boot
id_88.sp_1.m_rf.re_boot
id_89.sp_1.m_rf.re_boot
id_90.sp_1.m_rf.re_boot
id_91.sp_1.m_brt.re_boot
id_92.sp_1.m_brt.re_boot
id_93.sp_1.m_brt.re_boot
id_94.sp_1.m_brt.re_boot
id_95.sp_1.m_brt.re_boot
id_96.sp_1.m_brt.re_boot

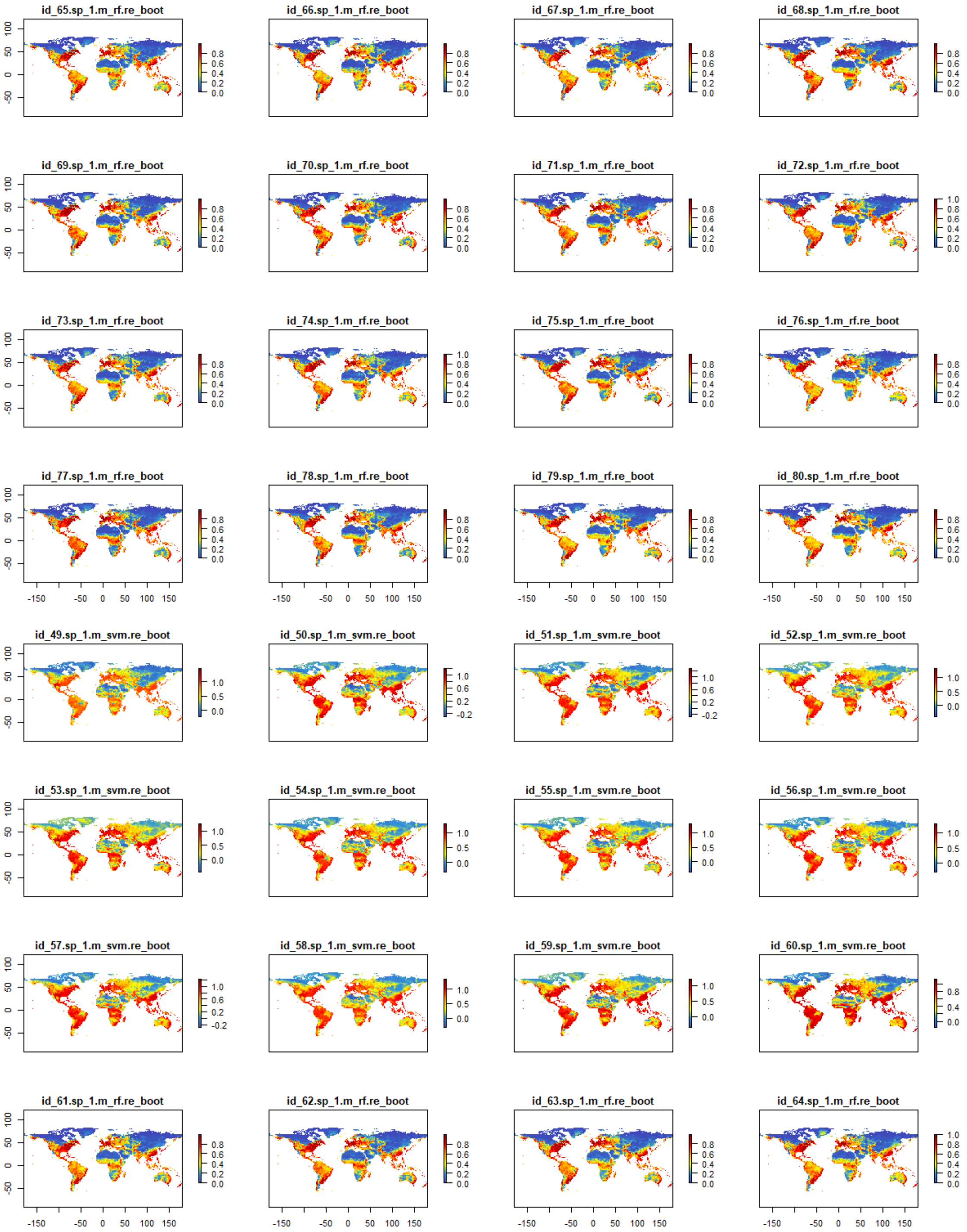
id_65.sp_1.m_rf.re_boot
id_66.sp_1.m_rf.re_boot
id_67.sp_1.m_rf.re_boot
id_68.sp_1.m_rf.re_boot
id_69.sp_1.m_rf.re_boot
id_70.sp_1.m_rf.re_boot
id_71.sp_1.m_rf.re_boot
id_72.sp_1.m_rf.re_boot
id_73.sp_1.m_rf.re_boot
id_74.sp_1.m_rf.re_boot
id_75.sp_1.m_rf.re_boot
id_76.sp_1.m_rf.re_boot
id_77.sp_1.m_rf.re_boot
id_78.sp_1.m_rf.re_boot
id_79.sp_1.m_rf.re_boot
id_80.sp_1.m_rf.re_boot
id_49.sp_1.m_svm.re_boot
id_50.sp_1.m_svm.re_boot
id_51.sp_1.m_svm.re_boot
id_52.sp_1.m_svm.re_boot
id_53.sp_1.m_svm.re_boot
id_54.sp_1.m_svm.re_boot
id_55.sp_1.m_svm.re_boot
id_56.sp_1.m_svm.re_boot
id_57.sp_1.m_svm.re_boot
id_58.sp_1.m_svm.re_boot
id_59.sp_1.m_svm.re_boot
id_60.sp_1.m_svm.re_boot
id_61.sp_1.m_rf.re_boot
id_62.sp_1.m_rf.re_boot
id_63.sp_1.m_rf.re_boot
id_64.sp_1.m_rf.re_boot

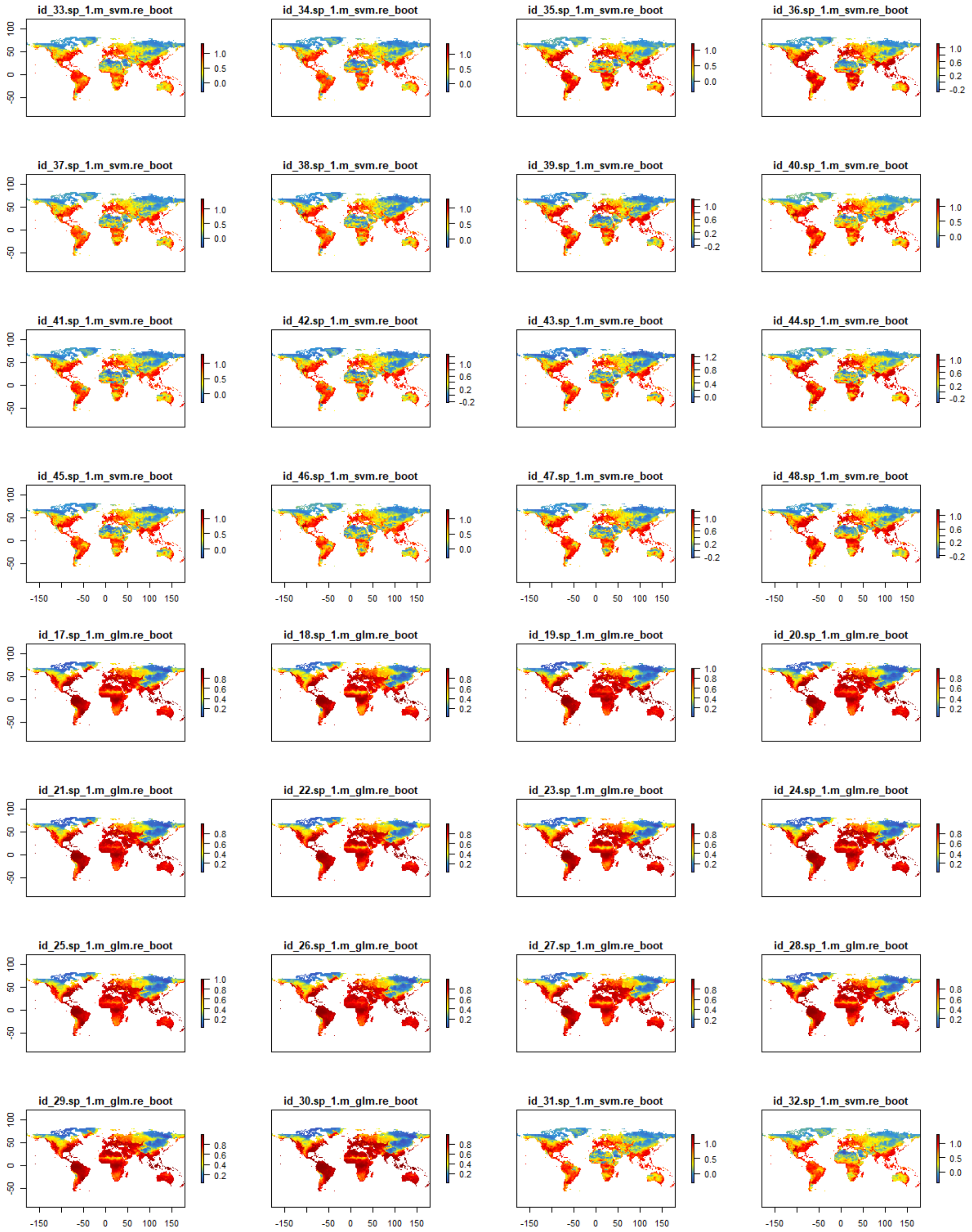
id_33.sp_1.m_svm.re_boot
id_34.sp_1.m_svm.re_boot
id_35.sp_1.m_svm.re_boot
id_36.sp_1.m_svm.re_boot
id_37.sp_1.m_svm.re_boot
id_38.sp_1.m_svm.re_boot
id_39.sp_1.m_svm.re_boot
id_40.sp_1.m_svm.re_boot
id_41.sp_1.m_svm.re_boot
id_42.sp_1.m_svm.re_boot
id_43.sp_1.m_svm.re_boot
id_44.sp_1.m_svm.re_boot
id_45.sp_1.m_svm.re_boot
id_46.sp_1.m_svm.re_boot
id_47.sp_1.m_svm.re_boot
id_48.sp_1.m_svm.re_boot
id_17.sp_1.m_glm.re_boot
id_18.sp_1.m_glm.re_boot
id_19.sp_1.m_glm.re_boot
id_20.sp_1.m_glm.re_boot
id_21.sp_1.m_glm.re_boot
id_22.sp_1.m_glm.re_boot
id_23.sp_1.m_glm.re_boot
id_24.sp_1.m_glm.re_boot
id_25.sp_1.m_glm.re_boot
id_26.sp_1.m_glm.re_boot
id_27.sp_1.m_glm.re_boot
id_28.sp_1.m_glm.re_boot
id_29.sp_1.m_glm.re_boot
id_30.sp_1.m_glm.re_boot
id_31.sp_1.m_svm.re_boot
id_32.sp_1.m_svm.re_boot

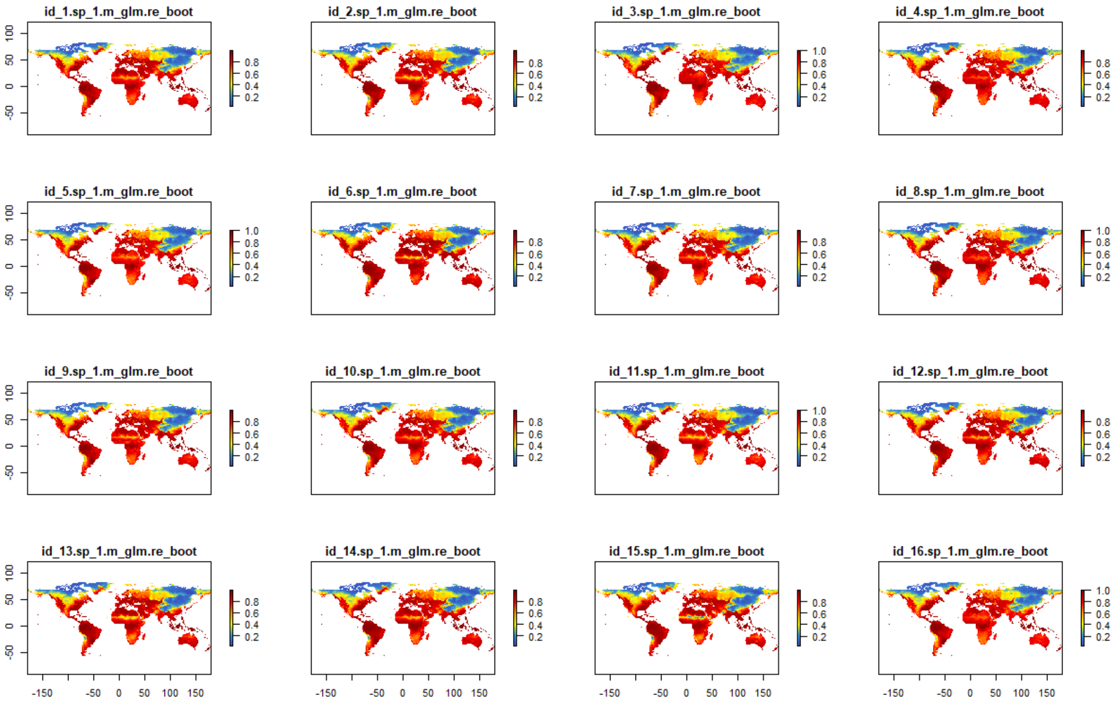
id_1.sp_1.m_glm.re_boot
id_2.sp_1.m_glm.re_boot
id_3.sp_1.m_glm.re_boot
id_4.sp_1.m_glm.re_boot
id_5.sp_1.m_glm.re_boot
id_6.sp_1.m_glm.re_boot
id_7.sp_1.m_glm.re_boot
id_8.sp_1.m_glm.re_boot
id_9.sp_1.m_glm.re_boot
id_10.sp_1.m_glm.re_boot
id_11.sp_1.m_glm.re_boot
id_12.sp_1.m_glm.re_boot
id_13.sp_1.m_glm.re_boot
id_14.sp_1.m_glm.re_boot
id_15.sp_1.m_glm.re_boot
id_16.sp_1.m_glm.re_boot

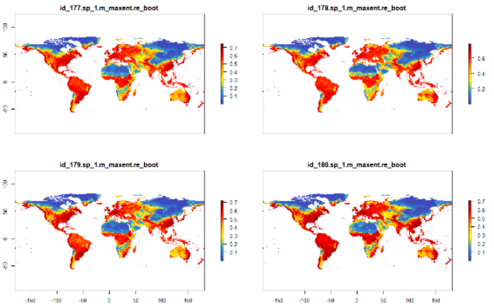
id_177.sp_1.m_maxent.re_boot
id_178.sp_1.m_maxent.re_boot
id_179.sp_1.m_maxent.re_boot
id_180.sp_1.m_maxent.re_boot

Supplementary material II. Response curves of bioclimatic variables

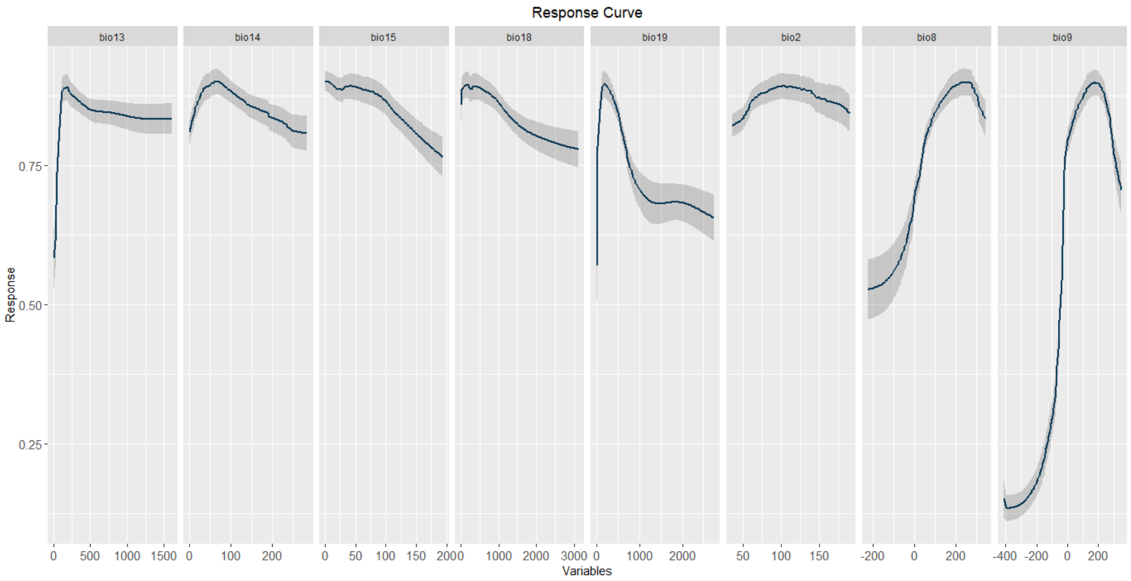

Supplement: Supplementary material 3 — Appendix S2 [file bdj-10-e90146-s003.pdf]
