# Supplementary material for: How could climate change influence the distribution of the black soldier fly, Hermetiaillucens (Linnaeus) (Diptera, Stratiomyidae)?
Source: Biodivers Data J. 2022 Oct 17;10:e90146. doi: 10.3897/BDJ.10.e90146 (PMC9836546; doi:10.3897/BDJ.10.e90146)
Supplement: Supplementary material 4 — Appendix S3 [file bdj-10-e90146-s004.pdf]

```
##SDM script for Hermetia portential distribution####

install.packages("sp")

install.packages("rgeos")

install.packages("raster")

install.packages("usdm")

install.packages("mapview")

library(sp)

library(rgeos)

library(raster)

write.table(sp,"XXXXX", sep=";")

sp<-read.csv("XXXX",sep=";",header=TRUE)

class(sp)

is.na(sp)

coordinates(sp)<- ~longitude + latitude

class(sp)

head(sp)

sp$name<-1

bio<-raster::getData("worldclim", var= "bio", res=2.5)

bio

library(usdm)

v1<-vifstep(bio)

v2<-vifcor(bio,th=0.7)

v1

v2

biom<-exclude(bio,v2)

biom

plot(biom[[1]])

points(sp,cex=0.5,pch=16)

mapview(sp)

library(sdm)

installAll()
```

```
head(sp)

d<- sdmData(name~., sp, predictors=biom, bg= list(n=1000))

d

m<- sdm(name~ . , d, methods=c("svm","rf","brt", "mars","maxent"),
replication=c("boot"),n=30)

m

gui(m)

p<-predict(m,biom,"predictions.img", overwrite=TRUE)

plot(p[[c(1:30)]])plot(p[[c(17:32)]],col=cl(200))

plot(p[[c(33:48)]])

plot(p[[c(49:64)]])

plot(p[[c(65:80)]])

plot(p[[c(81:96)]])

plot(p[[c(97:112)]])

plot(p[[c(113:128)]])

plot(p[[c(129:144)]])

plot(p[[c(145:160)]])

plot(p[[c(161:176)]])

plot(p[[c(177:180)]])

plot(en)

points(sp,cex=0.5,pch=16)

en2<-ensemble(m,biom," ",

          setting=list(method=" ",stat=" ",opt= ))

plot(en2)

#----------future-------

biof<-raster::getData("CMIP6",var="bio",res= ,rcp=" ",year=" ",model=" ")

names(biof)

names(bio)

names(biof)<-names(bio)

pf<-predict(m,biof, "predictionsf.img",overwrite=TRUE)
```

```
plot(pf[[c(1:16)]])
plot(pf[[c(17:32)]])
plot(pf[[c(33:48)]])
plot(pf[[c(49:64)]])
plot(pf[[c(65:80)]])
plot(pf[[c(81:96)]])
plot(pf[[c(97:112)]])
plot(pf[[c(113:128)]])
plot(pf[[c(129:144)]])
plot(pf[[c(145:160)]])
plot(pf[[c(161:176)]])
plot(pf[[c(177:180)]])

en2f<-ensemble(m,biof,"ensf2.img",
        setting=list(method=" ",stat=" ",opt= ))
plot(stack(en2,en2f))
plot(en2f)

#calculate the changes between the generated maps
ch<-en2f-en2
ch
plot(ch)

#presence and absence maps
ev<-getEvaluation(m,stat =c( " "," ","threshold"),opt= )
mean(ev$threshold)
pa<-raster(en2)
pa[]<-ifelse(en2[]>=XXXX,1,0)
plot(pa)
```
